# Pharmacokinetics of Active Components of Yokukansan, a Traditional Japanese Herbal Medicine after a Single Oral Administration to Healthy Japanese Volunteers: A Cross-Over, Randomized Study

**DOI:** 10.1371/journal.pone.0131165

**Published:** 2015-07-07

**Authors:** Hiroyuki Kitagawa, Masaya Munekage, Kengo Ichikawa, Ian Fukudome, Eri Munekage, Yuka Takezaki, Takashi Matsumoto, Yasushi Igarashi, Haruo Hanyu, Kazuhiro Hanazaki

**Affiliations:** 1 Department of Surgery, Kochi Medical School, Kochi University, Kochi, Japan; 2 Department of Surgical Oncology, Gifu University School of Medicine, Gifu, Japan; 3 Tsumura Research Laboratories, Kampo Scientific Strategies Division, Tsumura & Co., Ibaraki, Japan; 4 Kampo Formulations Development Center, Kampo Scientific Strategies Division, Tsumura & Co., Ibaraki, Japan; 5 Department of Elderly General Medicine, Tokyo Medical University, Tokyo, Japan; Johns Hopkins School of Medicine, UNITED STATES

## Abstract

**Context:**

Yokukansan (YKS) is a traditional Japanese herbal medicine called kampo medicine in Japan. Its extract comprises seven crude drugs: *Atractylodis lanceae rhizoma*, *Poria*, *Cnidii rhizoma*, *Uncariae uncis cum ramulus*, *Angelicae radix*, *Bupleuri radix*, and *Glycyrrhizae radix*. YKS is used to treat neurosis, insomnia, as well as behavioral and psychological symptoms of dementia.

**Objective:**

To confirm the exposure and pharmacokinetics of the active components of YKS in healthy volunteers.

**Design, Setting, and Participants:**

A randomized, open-label, 3-arm, 3-period, crossover trial was conducted on 21 healthy Japanese volunteers at the Kochi Medical University between May 2012 and November 2012.

**Interventions:**

Single oral administration of YKS (2.5 g, 5.0 g, or 7.5 g/day) during each period.

**Main Outcome Measure:**

Plasma concentrations of three active compounds in YKS, namely 18β-glycyrrhetinic acid (GA), geissoschizine methyl ether (GM), and hirsuteine (HTE).

**Results:**

The mean maximum plasma concentrations (C_max_) of GM and HTE increased dose-dependently (ranges: 0.650–1.98 ng/mL and 0.138–0.450 ng/mL, respectively). The times to maximum plasma concentration after drug administration (*t*_max_) were 0.500 h for GM and 0.975–1.00 h for HTE. The apparent elimination half-lives (*t*_1/2_) were 1.72–1.95 h for GM and 2.47–3.03 h for HTE. These data indicate the rapid absorption and elimination of GM and HTE. On the other hand, the *C*_max_, *t*_max_, and *t*_1/2_ of GA were 57.7–108 ng/mL, 8.00–8.01 h, and 9.39–12.3 h, respectively.

**Conclusion:**

We demonstrated that pharmacologically active components of YKS are detected in humans. Further, we determined the pharmacokinetics of GM, HTE, and GA. This information will be useful to elucidate the pharmacological effects of YKS.

**Trial Registration:**

Japan Pharmaceutical Information Center JAPIC CTI-121811

## Introduction

Yokukansan (YKS) is a traditional Japanese herbal medicine called kampo medicine in Japan. YKS is composed of seven crude drugs: *Atractylodis lanceae rhizoma*, *Poria*, *Cnidii rhizoma*, *Uncariae uncis cum ramulus*, *Angelicae radix*, *Bupleuri radix*, and *Glycyrrhizae radix*. It is approved by the Ministry of Health, Labour and Welfare as a prescription drug for the relief of symptoms of neurosis, insomnia, and night crying, and peevishness in children.

Several randomized studies have reported that YKS may be effective to treat behavioral and psychological symptoms of dementia caused by Alzheimer disease and dementia with Lewy bodies [1–3]. Recently, several basic studies have clarified that the glutamatergic [4–6] and serotonergic nervous [7–9] systems are associated with the mechanisms of YKS. Furthermore, the active components in YKS are also being studied. The improving effects of YKS on aggressive behavior and social behavior are mediated by the partial agonist activity on 5-HT_1A_ receptors, and representative active comoponents for these effects have been suggested to be geissoschizine methyl ether (GM) and hirsuteine (HTE) derived from *Uncariae uncis cum ramulus* [7, 8]. YKS exerts the neuroprotective effect by reducing the extracellular glutamic acid level via the activation of a glutamate transporter located on astrocytes, and *Glycyrrhizae radix*-derived 18β-glycyrrhetinic acid (GA) is suggested to be the active component responsible for this effect [6]. It is of great importance to reveal the pharmacokinetics (PK) of active components after administration in the efficacy and safety assessments of YKS. To date, a PK study was conducted in rats subjected to a single oral administration of YKS, and it was shown that GM, HTE, and GA are not only absorbed into the blood but also reach the brain, the site of drug action [10–12]. However, the PK of these three components after YKS administration is yet to be studied in humans.

In the present study, we enrolled 21 healthy adult Japanese volunteers to explore the possible mechanisms by which pharmacologically active YKS components are absorbed into the body and exert medicinal effects. As targets, we chose three YKS-derived components (GM, HTE, and GA) by referring to the results of the pharmacological and PK studies mentioned above. Further, we determined PK of GM, HTE, and GA in humans who were orally administered YKS.

## Methods

### Chemicals and reagents

**Fig 1:**
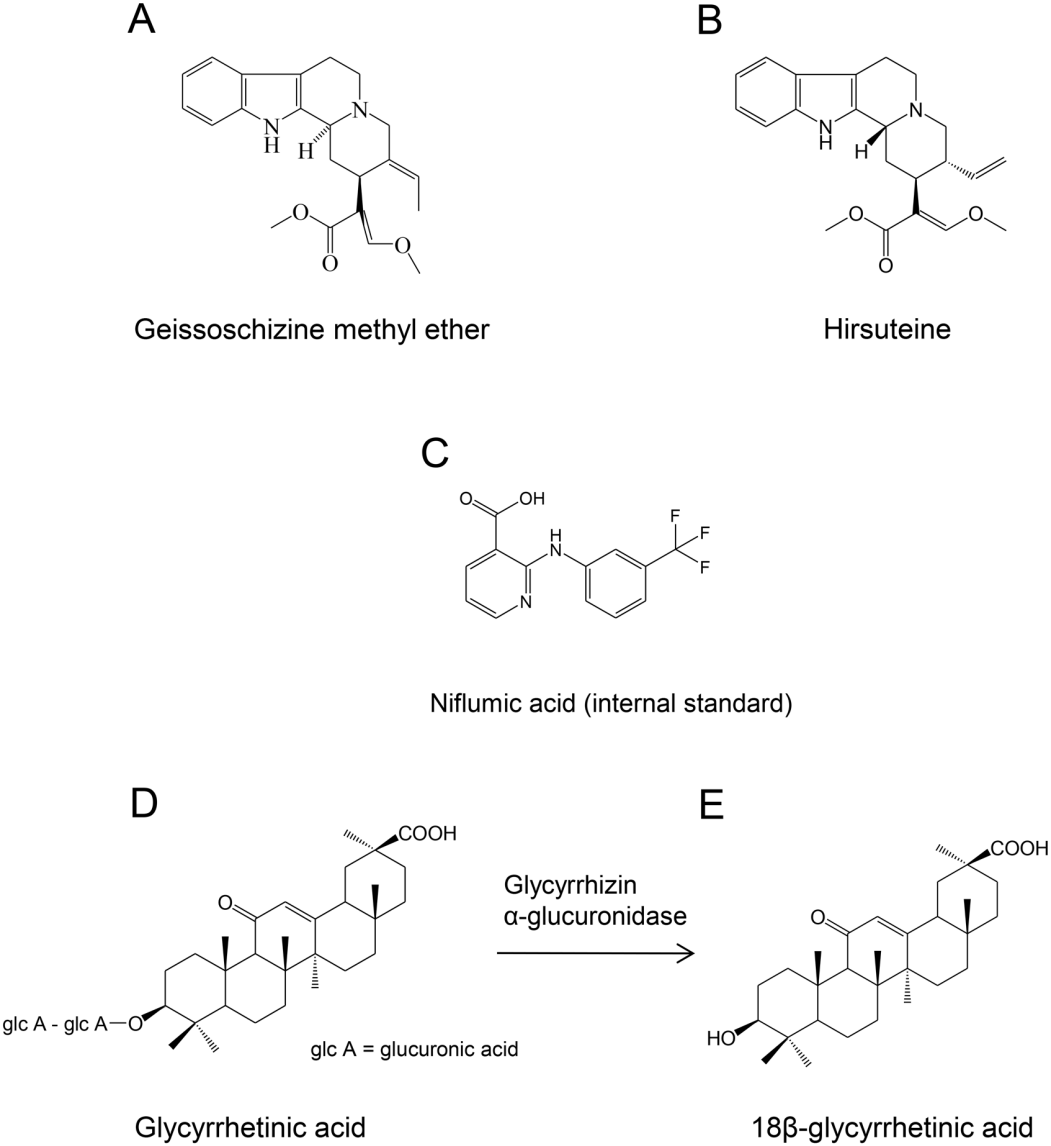
Chemical structures of yokukansan components, and metabolic process of glycyrrhizic acid. A; geissoschizine methyl ether, B; hirsuteine, C; niflumic acid (internal standard), D; glycyrrhizic acid, and E; 18β-glycyrrhetinic acid.

Tsumura Yokukansan Extract Granules for prescription (Product code; TJ-54, Tsumura & Co. Lot Number E43021, Tokyo, Japan) was used for the investigational product. It was manufactured according to GMP, and adapted to factory release test. The sample of the investigational drug used in this study is retained in Tsumura & Co. 7.5 g of this herbal preparation contains 3.25 g of dried extract obtained by spray drying a hot water extract of a mixture of seven crude drugs: 4.0 g of *Atractylodis lanceae rhizoma* (Asteraceae; atractylodes rhizome), 4.0 g of *Poria* (Polyporaceae; tuckahoe mushroom), 3.0 g of *Cnidii rhizoma* (Umbelliferae; cnidium root), 3.0 g of *Uncariae uncis cum ramulus* (Rubiaceae; Uncaria Hook), 3.0 g of *Angelicae radix* (Apiaceae; Chinese angelica root), 2.0 g of *Bupleuri radix* (Umbelliferae; bupleurum root), and 1.5 g of *Glycyrrhizae radix* (Fabaceae; Chinese liquorice root). The contents of GM, HTE, and glycyrrhizic acid (GL: a glycoside from GA that is metabolized to GA by intestinal bacteria *in vivo* [13]) in 1 g of YKS were 26.0, 21.5, and 2420 μg respectively, by liquid chromatography–mass spectrometry with tandem mass spectrometry analysis. GM, HTE, GL, and GA were supplied by Tsumura & Co. Liquid chromatography–mass spectrometry-grade acetonitrile and guaranteed grade ammonium acetate were purchased from Kanto Chemical Industry Co. Ltd. (Tokyo, Japan). Niflumic acid, which was used for internal standard was purchased from Sigma-Aldrich Co. (St. Louis, MO, USA). Water was purified using a pure-water supply system (Auto Pure WR600G, Yamato Scientific Co. Ltd. Tokyo, Japan). Other chemicals were purchased from commercial sources. Fig 1 shows the structures of GM, HTE, GL, GA, and niflumic acid.

### Ethics statement

The present study was conducted at the Kochi Medical School between May 2012 and November 2012 and was approved by the Ethical Committee Kochi Medical School. Furthermore, the study was conducted in accordance with ethical norms prescribed in the Declaration of Helsinki and good clinical practice guidelines. All subjects read and signed the informed consent form prior to entering the study.

### Clinical trial design

**Fig 2:**
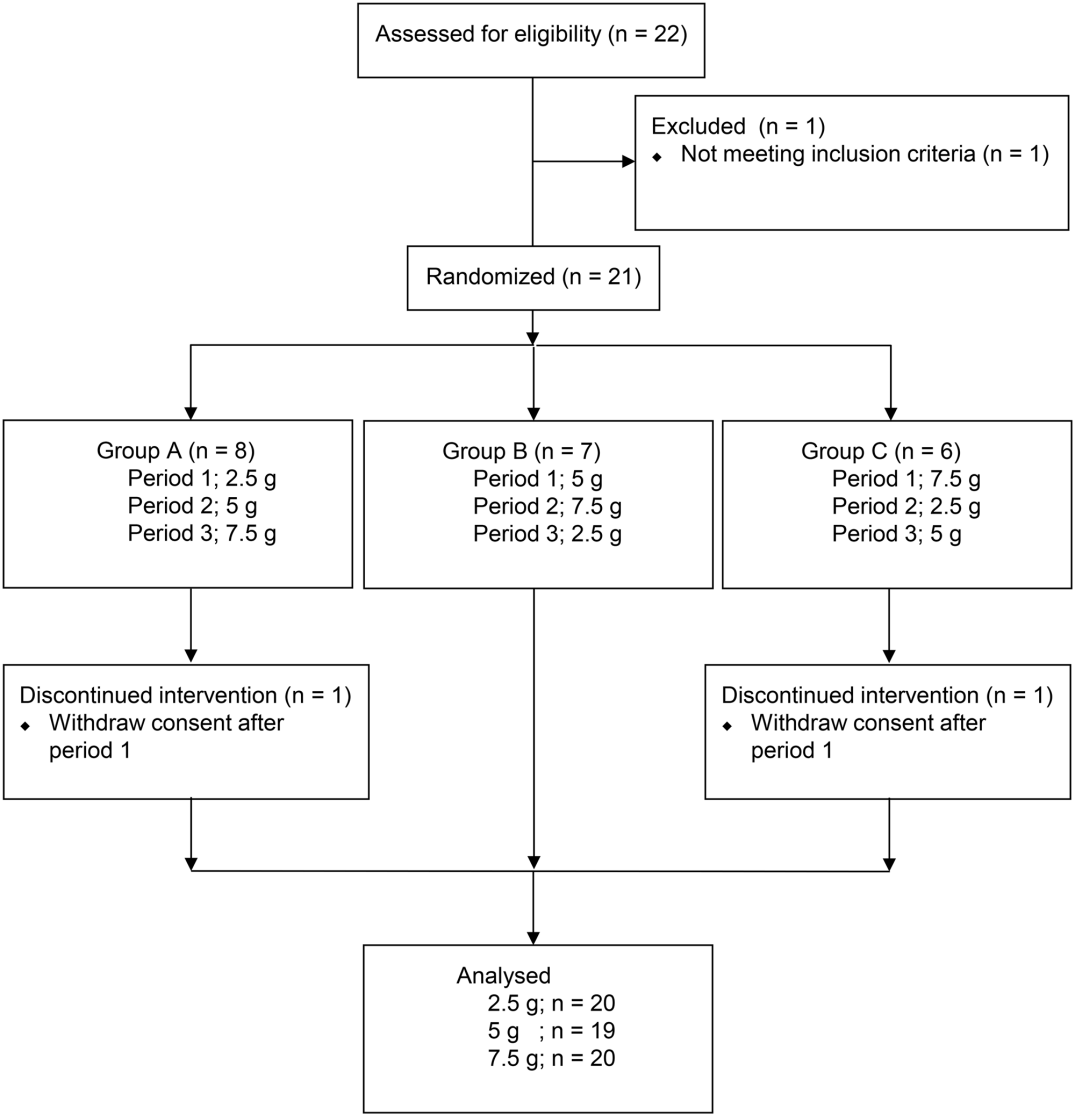
Flow diagram of participants through the study.

The present study was a randomized, open-label, three-arm, three-period design (Fig 2). The *t*_1/2_ of GA was longer than the other two compounds, and the *t*_1/2_ was previously reported using rats was approximately 7 h [12, 14]. In terms of the *t*_1/2_ of the three compounds measured in this study, a washout period of longer than five half-lives was adopted, and considering the load placed on subjects, it was set at four weeks or longer. Inclusion criteria included healthy Japanese adults between 20 and 45 years of age with a body mass index between 18 and 25 and who were willing and able to comply with the study requirements. Exclusion criteria included a history of significant liver, heart, or vessel disease and consumption of supplements that contained any YKS ingredients or any drug within 3 days to 1 week before the first dose. Other standard exclusion criteria included concerned allergies, pregnant or nursing females, and any alcohol or nicotine use. A sample size was chosen based on feasibility to allow characterization of the safety and PK of GM, HTE, and GA in healthy Japanese volunteers. Subjects allocated screening test were randomized to one of three groups by the central allocation system (allocation ratio 1:1:1). The random sequence was generated by computer in an independent center. The safety endpoint was evaluated in all volunteers. The safety endpoint was deemed to be based on the doctor’s determination, after examination or observation, to investigate if a serious adverse event had occurred. Adverse events included death, a life-threatening event, an event requiring hospitalization for treatment or an extended stay in hospital, an event resulting in permanent or temporary disability or dysfunction, an event resulting in a congenital abnormality, or any other serious medical phenomenon. Furthermore, in terms of side effects, no specific conditions were delineated, and all adverse events, in which it is impossible to deny a causal relationship with the drug, were deemed to be side effects. Therefore, at each stage during the 48-h period from the time of YKS administration, the doctor monitored the patient for subjective symptoms, objective findings, swelling, body temperature (axillary), blood pressure (sitting), pulse (sitting). The doctor also conducted a hematological examination to measure the patient’s red blood cell, leucocyte, and platelet counts, hemoglobin level, and hematocrit value. A biochemical examination was conducted to determine total protein, blood urea nitrogen, creatinine, uric acid, aspartate aminotransferase, alanine aminotransferase, total bilirubin, alkaline phosphatase, γ-glutamyltranspeptidase albumin, prothrombin time, total cholesterol, C-reactive protein and potassium, in order to determine any abnormalities compared with the subject condition prior to administration. All participants fasted for 12 h before administration and 4 h after administration of the study drug. The subjects were provided with meals that did not comprise any food or drink known to contain GM, HTE, or GL from three days before the start of the study to the day of its completion. Blood samples (8 mL each) were collected from subjects’ forearm cutaneous vein via a blood sampling tube that contained heparin at 0 (preadministration), 0.25, 0.5, 1, 2, 3, 4, 8, 10, 12, 14, 24, and 48 h after a single oral administration of YKS (2.5, 5.0, or 7.5 g). The trial participants were only hospitalized overnight on the day they ingested YKS, and they returned home the following day after blood samples were collected at time point of 24 h. The participants returned to the hospital to provide blood samples at time point of 48 h. The blood was immediately centrifuged at 1700 × *g* for 10 min to obtain the plasma. The plasma samples were stored at −20°C until analysis.

### Sample preparation

For quantification of GM and HTE, 300 μL of the plasma sample was mixed with 15 μL of water and acetonitrile (1:1, v/v), 15 μL of internal standard solution (500 ng/mL niflumic acid), and 1.2 mL of purified water. The mixture was loaded onto an Oasis HLB column (60 mg/3 mL cartridge: Waters Corporation, Milford, MA, USA), following it was washed with 3 mL of purified water and eluted with 2 mL of methanol. The eluate was dried at 40°C in a water bath under a stream of nitrogen gas. The residue was resolved with 150 μL of a solution of acetonitrile and 10 mmol/L ammonium acetate (1:2, v/v).

For quantification of GA, 500 μL of the plasma sample was mixed with 50 μL of 20% ethanol, 20 μL of internal standard solution (500 ng/mL niflumic acid), 250 μL of 3 M sodium acetate (pH 5.2) and purified water (1:9, v/v), and 4 mL of diethyl ether. The mixture was centrifuged at 1800 × *g* at 4°C for 3 min. The aqueous layer was frozen in a dry ice and methanol bath. The supernatant was collected in a test tube and dried at 40°C in a water bath under a stream of nitrogen gas. Thereafter, 200 μL of a mixture of acetonitrile and 10 mmol/L ammonium acetate (1:2, v/v) was added to the dried sample and mixed.

### Analysis conditions

*Analysis of GM and HTE*: The samples were chromatographically separated using Waters Acquity UPLC system (Waters Corporation), with an Acquity UPLC BEH C18 column (50 × 2.1 mm i.d., 1.7 μm particle size; Waters Corporation) at 40°C. The mobile phase consisted of solution A (10 mmol/L ammonium acetate) and solution B (acetonitrile) with a gradient of solution B (5%, 0 min; 5%, 0.2 min; 30%, 1.0 min; 30%, 4.4 min; 95%, 4.5 min; 95%, 5.0 min; v/v) at a flow rate of 0.6 mL/min. A 4000QTRAP triple quadrupole mass spectrometer fitted with a TurboIonSpray electrospray ionization interface (AB Sciex, Tokyo, Japan) was used for mass spectrometry and detection. The mass spectrometer was operated in positive-ion mode. The high-purity nitrogen gas was composed of ion source gas 1, ion source gas 2, curtain gas and collision-activated dissociation gas at pressures of 40, 40, 20, and 10 psi, respectively. The optimized TurboIonSpray voltage and temperature were set at 5000 V and 650°C, respectively. Both GM and HTE were quantified by multiple reaction monitoring transitions at 367.1 to 144.1 *m/z*.

*Analysis of GA*: The Shimadzu HPLC system (Shimadzu Corporation, Kyoto, Japan) consisted of two LC-10ADvp delivery pumps, a DGU-14A vacuum degasser, a SIL-HTc auto sampler and a CTO-10ASvp column oven. An InertSustain C18 column (50 × 2.1 mm i.d., 3 μm particle size; GL Sciences Inc., Tokyo, Japan) was used at 40°C. The mobile phase consisted of solution A (10 mmol/L ammonium acetate) and solution B (acetonitrile) with a gradient of solution B (20%, 0 min; 20%, 0.1 min; 65%, 2.2 min; 95%, 2.4 min; 95%, 3.1 min; v/v) at a flow rate of 0.5 mL/min. An API4000 triple quadrupole mass spectrometer fitted with a TurboIonSpray electrospray ionization instrument (AB Sciex) was used for mass spectrometry and detection. The mass spectrometer was operated in negative-ion mode. The high-purity nitrogen gas was composed of ion source gas 1, ion source gas 2, curtain gas, and collision-activated dissociation gas at pressures of 30, 50, 15, and 12 psi, respectively. The optimized TurboIonSpray voltage and temperature were set at −4200 V and 650°C, respectively. GA was quantified by multiple reaction monitoring transitions at 469.3 to 425.6 *m/z*.

### Method validation

The validation was conducted in accordance with both the US Food and Drug Administration’s “Guidance for Industry: Bioanalytical Method Validation” (2001) and the European Medicines Agency’s “Guideline on Validation of Bioanalytical Methods” (2011). The validation of this procedure was conducted in human plasma to evaluate the method in terms of specificity, recovery, intraday reproducibility, interday reproducibility, calibration curve, stability in blood, short-term stability, postpreparative stability, freeze–thaw stability, long-term stability, dilution integrity, matrix effect, carry-over, and stability in the standard solution.

### Pharmacokinetics and statistical analysis

Plasma PK data were analyzed by non-compartment modeling using Phoenix WinNonlin (version 6.3, Certara L.P., St. Louis, MO, USA) to determine various PK constants, including maximum concentration (*C*_max_), time to maximum concentration (*t*_max_), apparent elimination half-line (*t*_1/2_) and area under the plasma concentration-time curve from zero to last observation time(AUC_*0-last*_). The elimination half-life (*t*_1/2_) was calculated divided by log_e_2/*k*e, where *k*e is the terminal elimination (at least three data points on the descending linear limb) rate constant. The plasma concentration, *C*_max_, AUC_0–last_, and *t*_1/2_ of the target components in each group are presented as the geometric mean and 95% confidence intervals. The *t*_max_ data are presented as the median with ranges from minimum to maximum. The dose proportionality was analyzed via a power model [15, 16] (eq 1):

$$\mu_{i}=\mu +\beta \;\cdot \text{ln}\left({\text{Dose}}_{i}\right)$$ (1)

μ_*i*_ is the natural logarithm of the AUC_*0–last*_ or *C*_max_ at dose (*i* = 1,2,3). The Dose_*i*_ is the administered dose (g) of the test drug. The model fitted as a linear mixed-effects model that included the random subject effect (eq 2):

$$\mu_{ij}=\mu_{i}+{\text{a}}_{j}+\varepsilon_{ij}$$ (2)

a_*j*_ is the random effect parameter that shows the individual difference for subject j, which is assumed to be normally distributed around mean 0 with variance σ_a_^2^. ε_*ij*_ is express of the random error with mean 0 and variance σ^2^. β is the parameter to be used for the dose proportionality evaluation, and μ is the intercept. Inferences were made based on the theoretical β of 1, and confidence limits of 0.8 and 1.25. Evaluation of linearity of the dosage-exposure relations analysis was conducted using Phoenix WinNonlin and SAS 9.1.3 (SAS Institute, Inc., Cary, NC).

## Results

### Registered subjects

Twenty two subjects were screened and one subject was excluded as not meeting the inclusion and exclusion criteria. Therefore, 21 subjects (13 men and 8 women) were enrolled for the present study. Table 1 shows demographics of the 21 subjects in each group. Two subjects were discontinued this study after first dosing since the visit schedule was not able to be adjusted. However the data from first dosing were included to the PK analysis. Fig 2 summarized flow diagram of participants. No adverse effect was observed in any subject treated with YKS.

**Table 1:** Demographics.

Group	n	Age	Height (cm)	Weight (kg)	BMI (kg/m^2^)	Male/Female
A	8	24 (20–32)	173.3 (158.7–184)	65.6 (47.7–82.6)	21.9 (18.9–24.7)	5/3
B	7	26 (24–29)	166.1 (145.8–176)	57.8 (39.4–77.4)	20.5 (18.5–25)	4/3
C	6	23.5 (22–33)	162.2 (152.7–175.9)	52.3 (44.5–64.5)	20.2 (18.5–22.3)	4/2

Data represent the median (minimum–maximum)

### Plasma concentrations of geissoschizine methyl ether, hirsuteine, and 18β-glycyrrhetinic acid after yokukansan administration

**Fig 3:**
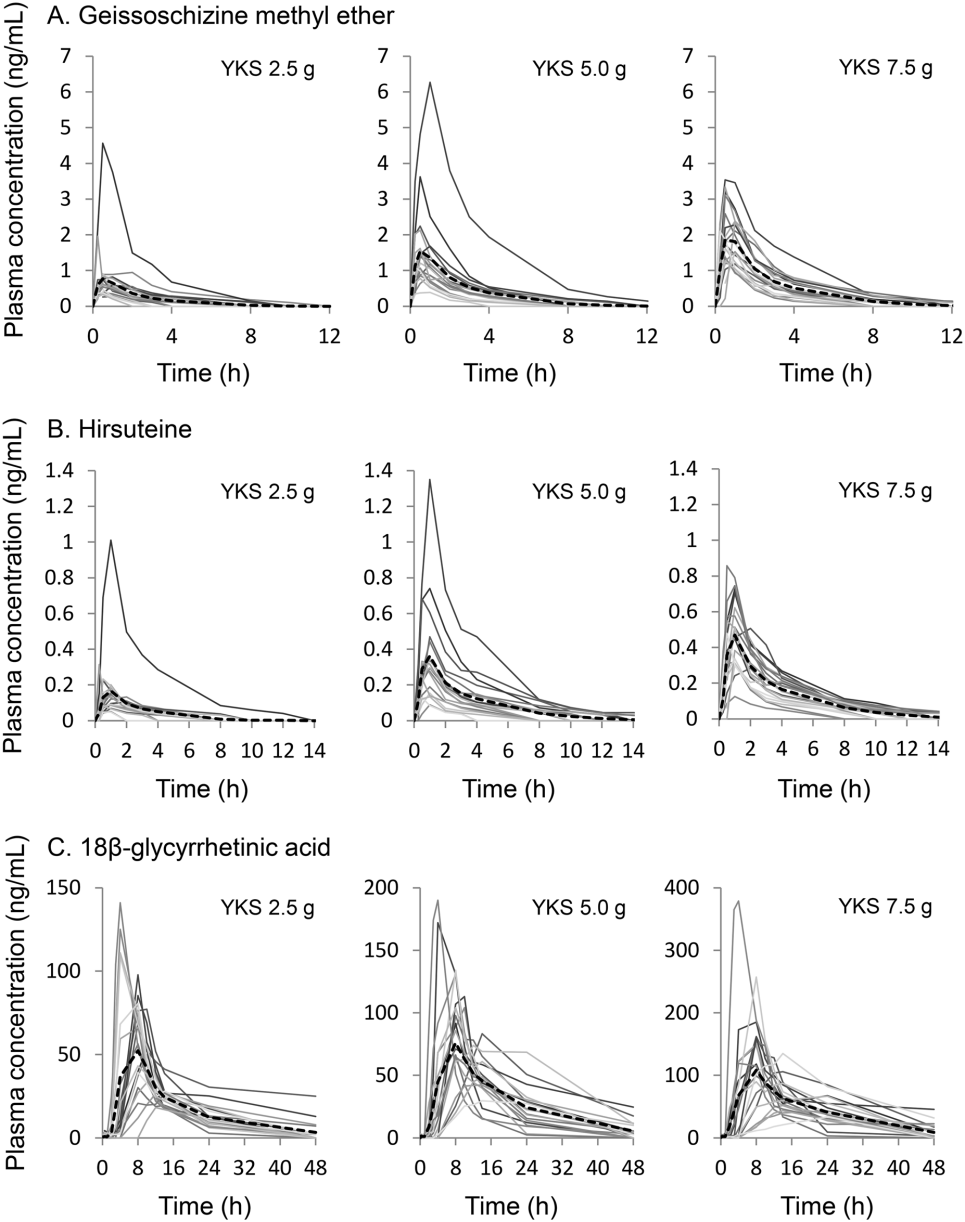
Plasma concentrations of geissoschizine methyl ether, hirsuteine, and 18β-glycyrrhetinic acid after oral administration of yokukansan. A; geissoschizine methyl ether, B; hirsuteine, and C; 18β-glycyrrhetinic acid. Blood samples were collected at 0 (beginning of the study), 0.25, 0.5, 1, 2, 3, 4, 8, 10, 12, 14, 24, and 48 h after administration of yokukansan (YKS, 2.5, 5.0, or 7.5 g/day). Line, plasma concentration–time profile of each subject; Dotted line, mean of the plasma concentration–time profile.

Individual plots and mean plot of plasma concentrations of GM, HTE, and GA against time at each of the 3 doses of YKS are shown in Fig 3. The PK parameters of the three components are shown in Table 2. Although a GA peak was detected in some subjects before YKS administration, the concentration in any of these subjects was close to the below quantification limit, and pharmacokinetic analysis of GA was conducted using data containing that information.

**Table 2:** Pharmacokinetic parameters of yokukansan components.

Parameter	2.5 g (*n* = 20)	5.0 g (*n* = 19)	7.5 g (*n* = 20)
	Geometric mean (95% Confidence interval)	Geometric mean (95% Confidence interval)	Geometric mean (95% Confidence interval)
Geissoschizine methyl ether			
*C*_max_, ng/mL	0.650 (0.480–0.880)	1.39 (1.04–1.85)	1.98 (1.68–2.33)
AUC*_0-last_*, ng·h/mL	1.18 (0.800–1.75)	2.98 (2.10–4.24)	4.81 (3.78–6.13)
*t*_1/2_, h	1.72 (1.40–2.12)	1.85 (1.59–2.16)	1.95 (1.68–2.28)
*t*_max_, h (median range)	0.500 (0.250–1.83)	0.500 (0.250–1.00)	0.500 (0.250–1.00)
Hirsuteine			
*C*_max_, ng/mL	0.138 (0.100–0.190)	0.305 (0.223–0.419)	0.450 (0.366–0.554)
AUC*_0-last_*, ng·h/mL	0.277 (0.180–0.427)	0.833 (0.534–1.30)	1.50 (1.16–1.95)
*t*_1/2_, h	2.47 (2.07–2.96)	2.97 (2.56–3.45)	3.03 (2.71–3.39)
*t*_max_, h (median range)	1.00 (0.250–1.85)	0.983 (0.467–1.00)	0.975 (0.500–2.00)
18β-glycyrrhetinic acid			
*C*_max_, ng/mL	57.7 (43.9–75.7)	84.3 (67.4–106)	108 (81.2–145)
AUC*_0-last_*, ng·h/mL	690 (563–845)	1210 (1010–1460)	1670 (1260–2200)
*t*_1/2_, h	11.0 (8.19–14.7)	9.39 (7.09–12.4)	12.3 (8.26–18.3)
*t*_max_, h (median range)	8.00 (3.85–13.8)	8.00 (3.85–13.8)	8.01 (4.00–23.8)

*t*_1/2_ of GM (2.5 g) and HTE (2.5 g) were *n* = 18. *t*_1/2_ of GA (7.5 g) was *n* = 17.

*C*_max_ of GM and HTE increased in a dose-dependent manner in the ranges of 0.650–1.98 ng/mL and 0.138–0.450 ng/mL, respectively, *t*_max_ was 0.500 h for GM and 0.975–1.00 h for HTE, and *t*_1/2_ was 1.72–1.95 h for GM and 2.47–3.03 h for HTE. The dose-dependent increases in plasma concentrations of GM and HTE were also confirmed by AUC_*0–last*_ values (1.18–4.81 ng·h/mL and 0.277–1.50 ng·h/mL in the 2.5–7.5 g groups).

The concentration of GA in the plasma was very high compared with the concentrations of GM or HTE. *C*_max_ increased in a dose-dependent manner (57.7, 84.3, and 108 ng/mL in the 2.5, 5.0, and 7.5 g groups, respectively) and *t*_max_ in each group was approximately 8 h (8.00, 8.00, and 8.01 h in the 2.5, 5.0, and 7.5 g groups, respectively). Thereafter, the concentration of GA in each group gradually reduced, and *t*_1/2_ was 11.0, 9.39, and 12.3 in the 2.5, 5.0, and 7.5 g groups, respectively. The AUC_*0–last*_ values were 690, 1210, and 1670 ng·h/mL in the 2.5, 5.0, and 7.5 g groups, respectively.

**Fig 4:**
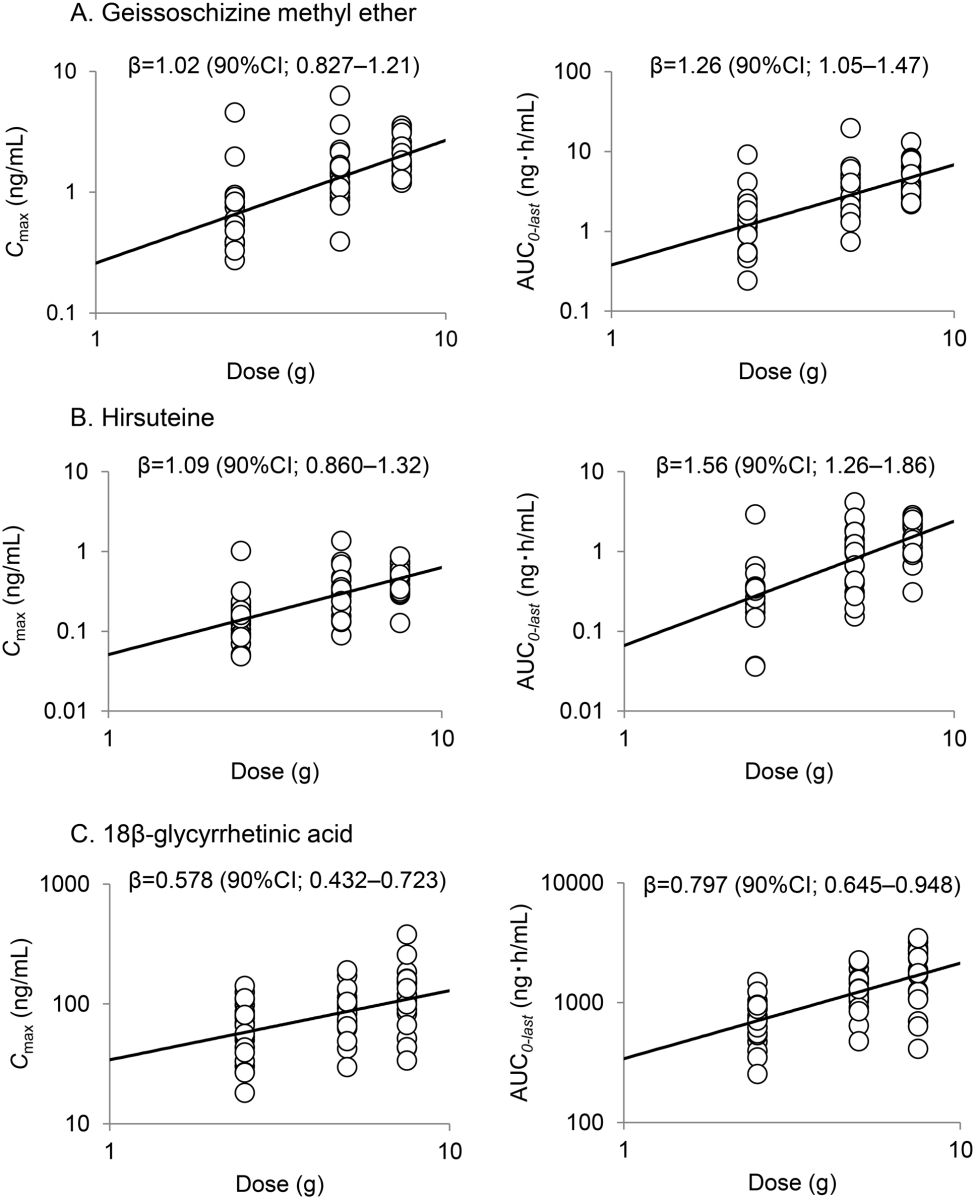
Relations between log-transformed dosage and *C*_max_ or AUC_*0–last*_. A; geissoschizine methyl ether, B; hirsuteine, and C; 18β-glycyrrhetinic acid. Power regression model was fitted to the mixed effect model for evaluation of linearity. CI; confidence interval.

The dose proportionality of *C*_max_ and AUC_*0–last*_ are displayed in Fig 4. The estimated β (90% confidence interval) of *C*_max_ for GM was 1.02 (0.827–1.21), and the 90% confidence intervals included 1 for doses ranging between 2.5 and 7.5 g of YKS. These results suggested that the *C*_max_ of GM was linear within the dose range of 2.5 to 7.5 g/day of YKS. However, the 90% confidence intervals of the β value of HTE and GA for *C*_max_ and GM, HTE, and GA for AUC_*0–last*_ did not include 1, and confidence limits were out of the range of 0.8–1.25. The estimate of the covariance parameter showed that the between-subject variability (σ_a_^2^ = 0.216) was larger than the random error variability (σ^2^ = 0.0889). This suggested that the crossover design was appropriate.

## Discussion

In the present randomized cross-over study, we determined PK of components GM, HTE, and GA in the plasma of humans who orally received YKS. We demonstrated that the candidate active components of YKS were absorbed into the blood at an effective dose in humans. This study provides information on YKS components which are exposed in human body, and linearity that were observed in PK data.

GM and HTE are indole alkaloid compounds present in *Uncariae uncis cum ramulus* and are believed to constitute a part of pharmacologically active components of *Uncariae uncis cum ramulus* [8]. However, no reports have shown changes in plasma levels of GM and HTE levels following YKS administration. The present study demonstrated that GM and HTE were rapidly absorbed following YKS administration in humans. The dose proportionality of *C*_max_ for GM was linear with the dose, but HTE for *C*_max_ and GM and HTE for AUC_0–last_ did not show linearity. The *C*_max_ for the lowest dose of YKS (2.5g) group did not reach tenfold of the limit of qualification. This may be considered that AUC_0–last_ was underestimated due to the truncation error, leading to the discontinuation of the measurement.

In rat models, GM and HTE have been shown to enter the brain when an effective dose is orally administered [12]. The plasma levels of GM and HTE in the abovementioned study were 0.3–2.0 ng/mL and 0.2–0.4 ng/mL, respectively, and are almost equivalent to the human plasma levels observed in the present study. GM and HTE, acting as partial agonists of 5-HT_1A_ receptors in the brain, are considered to be a part of the mechanism of action of YKS [8]. The present study confirmed the exposure in humans and supports the presumed mechanism of YKS action in humans.

GM and HTE are rapidly eliminated and have half-lives of 2–3 h. Although the elimination pathway is not yet clear, HTE reportedly is metabolized by CYP2C in rats [17]. GM is structurally very similar to HTE. Therefore, GM may also be metabolized by CYP. Because some humans are poor metabolizers of CYP2C [18] and it is an important factor that affects blood kinetics, metabolic enzymes of GM and HTE should be studied further in humans.

GA, another component measured in the present study, is a compound derived from *G*. *radix*, which is a plant widely used in medicines and foods around the world. GA is an aglycon of GL, a component of *G*. *radix*. GL is poorly absorbable and was not detected in the plasma of humans in the previous study [19]. However, GL is metabolized to GA by intestinal bacteria and is then absorbed into the blood [20]. GA *t*_max_ was substantially greater than that of GM and HTE in the present study. This is consistent that GA is first metabolized by enterobacteria in the intestinal tract and is then absorbed [20].

An adequate washout period was set in this trial and a GA peak had been detected in the plasma of some subjects in the initial trial. GA peak was detected in plasma before YKS administration in a number of subjects. As various foods and sweeteners are known to contain glycyrrhiza, which contains GL, a glycoside of GA, we regulated the meals of subjects during this study. However, it had not been completely excluded from the meals and represents a likely source of the trace GA detected in the plasma. Nevertheless, given the result that the GA level in plasma increased after YKS administration and then returned to the base level, it would be reasonable to conclude that the analysis method had sufficient specificity and was satisfactory for the PK analysis in the present study. Furthermore, the concentration was close to the below quantification limit, and it was believed that the peak was not affect pharmacokinetics of GA by YKS administration.

AUC of GA after YKS administration tended to be slightly larger than AUC of GA after other *Glycyrrhizae radix*-containing kampo medicines were administered to humans [21]. *Glycyrrhizae radix*, a crude drug containing GL, a glycoside of GA, is frequently used in foods and medicines, and various reports are available on the PK of *Glycyrrhizae radix* or GL administration [22–24]. However, the absorption and metabolism of kampo medicine components can be controlled by interactions among constituting crude drugs or components [14, 21, 25, 26]; therefore, it is of importance to reveal the PK of each component when they are administered as a kampo medicine or mixture of crude drugs rather than as a single component. The present study suggested that the PK of GA was altered when administered as YKS, and the underlying mechanism needs to be elucidated in the future.

Plant medicines are multicomponent drugs and exert a variety of effects. Therefore, it is difficult to interpret each component’s PK properties. In the present study, we measured components that are believed to contribute to the effect of YKS and found that PK varied greatly from component to component. Researchers should examine the PK/PD of YKS in future studies. Such studies will provide a scientific rationale for dosage and administration, both of which are currently defined based on empirical or practice standards, and will enable adequate medication guidance. The present study is a valuable contribution in these efforts.

In conclusion, we revealed that the pharmacologically active components of YKS are absorbed by the human body and in addition, determined the pharmacokinetics of GM, HTE, and GA. The information will be useful to the elucidation of possible pharmacological actions of YKS.

## Supporting Information

S1 Checklist CONSORT checklist. (DOC)

S1 Protocol Clinical trial protocol (Japanese version). (PDF)

S2 Protocol Clinical trial protocol (English version). (DOC)
