# Supplementary material for: Pharmacokinetics of Active Components of Yokukansan, a Traditional Japanese Herbal Medicine after a Single Oral Administration to Healthy Japanese Volunteers: A Cross-Over, Randomized Study
Source: PLoS One. 2015 Jul 7;10(7):e0131165. doi: 10.1371/journal.pone.0131165 (PMC4495062; doi:10.1371/journal.pone.0131165)
Supplement: S1 Protocol — (PDF) [file pone.0131165.s002.pdf]

TJ-54-4-3
Ver. 1.0
2011年12月27日作成

抑肝散（TJ－54）単回投与後の
健常人を対象とした薬物動態試験

製造販売後臨床試験実施計画書

Ver.1.0

2011年12月27日作成

試験依頼者	株式会社ツムラ　育薬企画部
	東京都港区赤坂二丁目17番11号<br>TEL. 03-6361-7185（直通）

目次

1. 試験概要 1
1.1 試験課題名 1
1.2 試験の目的 1
1.3 評価項目 1
1.4 対象 1
1.4.1 対象被験者 1
1.4.2 目標症例数 1
1.4.3 選択基準 1
1.4.4 除外基準 1
1.5 試験デザイン 2
1.6 投与方法および投与量 2
1.7 投薬の概要 2
1.8 その他の管理 2
1.9 試験期間 3
1.10 試験実施医療機関 3
1.11 試験依頼者 3
2. 経緯 4
3. 試験の目的 5
3.1 試験の目的 5
3.2 試験の種類 5
4. 対象 5
4.1 対象被験者 5
4.2 目標症例数 5
4.3 選択基準 5
4.4 除外基準 5
4.5 登録の方法 6
4.5.1 被験者選定 6
4.5.2 スクリーニング名簿/被験者登録名簿の作成 6

4.5.3 被験者識別コードリストの作成 7
4.5.4 被験者の割付 7
5. 試験方法 7
5.1 試験デザイン 7
5.2 投薬の概要 7
5.3 試験の完了および中止・脱落 8
5.3.1 完了 8
5.3.2 中止・脱落基準 8
5.3.3 中止手順 8
5.3.4 被験者の管理 8
6. 被験薬 9
6.1 使用薬名および製造元 9
6.2 成分、剤型および保管条件 9
6.3 投与方法および投与量 9
6.4 被験薬の交付 10
6.5 被験薬の保管および管理 10
6.6 被験薬の回収 10
7. 検査・観察項目および実施時期 10
7.1 検査・観察項目 10
7.1.1 スクリーニング検査項目 10
7.1.2 投与前検査 11
7.1.3 投与時検査 11
7.1.4 終了時検査 11
7.1.5 血漿中 TJ-54 由来成分の探索 12
7.1.6 安全性項目 13
7.2. 実施スケジュール 13
8. 評価の基準および解析 15
8.1 対象症例 15
8.1.1 薬物動態に関する解析症例 15

8.1.2　安全性に関する解析症例 15
8.2　評価項目 15
8.2.1　薬物動態 15
8.2.2　安全性評価 16
8.2.3　症例固定 16
8.3　統計解析計画 16
9.　有害事象 16
9.1　定義 16
9.1.1　有害事象 16
9.1.2　重篤な有害事象 16
9.1.3　副作用 17
9.1.4　被験薬との因果関係 17
9.1.5　有害事象の重症度 17
9.2　有害事象のモニタリング 18
9.2.1　発生時の対応 18
9.3　新たな情報の提供 18
9.4　予測される副作用 19
10.　倫理的事項 19
10.1　倫理原則の遵守 19
10.2　治験審査委員会 19
10.3　インフォームドコンセント 19
10.3.1　同意取得の時期と方法 19
10.3.2　同意説明文書による説明事項 19
10.3.3　同意説明文書の承認と改訂 20
10.4　個人情報の保護 21
10.5　金銭の支払い 21
10.6　健康被害補償および保険 21
10.7　試験の終了または中止および中断 21
10.7.1　試験の終了 21

10.7.2 試験全体の中止または中断の基準 22

10.7.3 試験中止または中断の手続き 22

11. 試験実施計画書の遵守および逸脱または変更ならびに改訂 22

11.1 試験実施計画書の遵守 22

11.2 試験実施計画書の逸脱または変更 22

11.3 実施計画書の改訂 22

12. データの収集および保存 23

12.1 症例報告書の作成 23

12.2 記録の保存 23

13. 原資料の直接閲覧 23

13.1 原資料の特定 23

13.2 直接閲覧の方法 24

13.3 閲覧による照合・確認結果の評価および対応 24

14. 試験の品質管理および保証 24

14.1 品質管理 24

14.2 品質保証 24

15. 公表に関する取り決め 24

16. 試験期間 24

17. 試験実施体制 25

17.1 試験依頼者 25

17.1.1 試験依頼者 25

17.1.2 試験依頼者の試験組織 25

17.1.3 メディカルアドバイザー 25

17.2 血漿および尿中 TJ-54 由来成分の分析施設 25

17.3 試験実施医療機関および試験責任医師 25

18. 参考文献 25

19. 統計解析責任者及びメディカルアドバイザー(医学専門家)の確認欄 28

20. 本試験実施計画書遵守の合意 29

1. 試験概要

1.1 試験課題名

健常人を対象とした抑肝散(TJ-54)の薬物動態試験

1.2 試験の目的

日本人健常成人を対象として、ツムラ抑肝散エキス顆粒(医療用)(TJ-54) 7.5g (1 包 2.5g×3 包)、 5.0 g (1 包 2.5g×2 包)、2.5g (1 包 2.5g×1 包)、の単回経口投与時における薬物動態および安全性について検討する。

1.3 評価項目

薬物動態および安全性

1.4 対象

1.4.1 対象被験者

選択基準にすべて合致し、除外基準に抵触しない健常成人

1.4.2 目標症例数

目標完了症例数として 15 例以上

1.4.3 選択基準

1) 日本人
2) 20 歳以上～45 歳未満の者(同意取得時)
3) 肥満度(BMI)が 18.5 以上 25 未満の者
4) 性別:不問
5) 本人から文書による同意を得た者

1.4.4 除外基準

1) 薬物アレルギー(漢方薬を含む)、食物アレルギーの既往歴のある者
2) 妊婦、妊娠を希望する者、授乳している者
3) 被験薬投与前 16 週以内に、他の臨床研究に参加した者
4) 被験薬投与前 12 週以内に 400mL を超える採血・献血を行った者
5) 重篤な肝疾患、心疾患および血液疾患のうちいずれかの既往歴のある者
6) 脂肪肝の者
7) 各期の被験薬投与の 3 日前から各期の終了まで、アルコールあるいはタバコを中止できない者

8) 各期の被験薬投与の 7 日前から各期の終了まで、薬剤(たとえば向精神薬、抗真菌薬、抗高血圧薬、漢方薬等)を中止できない者
9) カンゾウ、センキュウ、トウキ、サイコ、チョウトウコウ、ソウジュツ、ブクリョウを由来とするサプリメントを被験薬投与の 3 日以内に投薬している者
10) 各期の被験薬投与の 7 日前から被験薬投与までの間に、何らかの薬剤を投薬した者
11) スクリーニング検査または投与前検査において、臨床検査の基準範囲を超え、試験責任医師等が不適当と判断した者
12) その他、試験責任医師等が不適当と判断した者
13) HCV 抗体、HBs 抗原、HIV 抗体陽性のもの

1.5 試験デザイン

ランダム化クロスオーバー試験

1.6 投与方法および投与量

ツムラ抑肝散エキス顆粒(医療用)7.5g (1 包 2.5g×3 包)、 5.0 g (1 包 2.5g×2 包)、2.5g (1 包 2.5g×1 包)を単回経口投与する。

1.7 投薬の概要

被験者を登録時に A～C 群に分け、同一被験者に対して 3 用量を 3 回に分けて投与する。3 回の投与時期は 1 期、2 期、3 期とし、1 期と 2 期および 2 期と 3 期の間隔は 4 週月以上のウォッシュアウト期間を設ける(表 1)。

表 1 投薬の概要

	1 期	ウォッシュアウト期間	2 期	ウォッシュアウト期間	3 期
A 群	7.5g 投与	4 週以上	2.5g 投与	4 週以上	5.0g 投与
B 群	2.5g 投与	4 週以上	5.0g 投与	4 週以上	7.5g 投与
C 群	5.0g 投与	4 週以上	7.5g 投与	4 週以上	2.5g 投与

1.8 その他の管理

1) 各期の被験薬投与の 3 日前から各期の終了まで、カフェイン、アルコール、炭酸水および甘草含有飲食物〔原材料名に標記があるもの〕、にんにく、柑橘類(グレープフルーツ、オレンジ、みかん等)を含む飲食品(香辛料を含む)〔別紙参照〕ならびに抑肝散の構成生薬(カンゾウ、センキュウ、トウキ、サイコ、チョウトウコウ、ソウジュツ、ブクリョウ)を由来とするサプリメントの摂取を禁止する。ただし、飲料水の摂取は適宜自由とする。被験薬投与前日 20:00 以降から投与後 4 時間までは絶食とする。

2) 喫煙：各期の被験薬投与の 3 日前から各期の終了まで、禁煙とする。

3) 他剤の使用：被験薬投与の 7 日前から各期の試験終了まで他剤の使用を禁止する。

1.9　試験期間

2012 年 5 月～2012 年 12 月

1.10 試験実施医療機関

高知大学医学部附属病院

〒783-8505 高知県南国市岡豊町小蓮

TEL:088-866-5811

1.11 試験依頼者

株式会社ツムラ 〒107-8521 東京都港区赤坂二丁目 17 番 11 号

連絡先:育薬企画部

TEL:03-6361-7185　FAX:03-5574-6664

2. 経緯

抑肝散は、漢方の古典(保嬰撮要)に記載されている薬方であり、ソウジュツ、ブクリョウ、センキュウ、チョウトウコウ、トウキ、サイコおよびカンゾウの 7 種の生薬より構成される漢方薬である。古来より「虚弱な体質で神経がたかぶるもの」に対し広く用いられてきた。ツムラ抑肝散エキス顆粒(医療用)(TJ-54)は、TJ-54 をツムラ独自の乾式造粒法により服用しやすい顆粒剤として製剤化し、これを「厚生省薬務局薬審第二第 120 号通知(昭和 60 年 5 月 31 日付)」に基づき製造承認申請し、承認された医療用漢方エキス製剤である。TJ-54 は不眠[1)]、小児疳症、認知症の行動・心理症状[2)~5)]に広く用いられている。薬理作用として、攻撃性改善作用[6)~9)]が報告されている。また、その作用機序としてセロトニン神経系[9)~11)]、グルタミン酸神経系[12)~15)]などへの関与が報告されている。

一方、薬物動態に関する研究については、構成生薬での研究報告は散見されるものの、極めて多くの成分を含むために、これまで TJ-54 としての薬物動態研究は実施されてこなかった。構成生薬の甘草については、ヒトへのカンゾウエキス顆粒剤(カンゾウエキス粉末約 0.63g、グリチルリチン酸として 60.2mgを含む)投与後のグリチルレチン酸(グリチルリチン酸代謝物)を定量した研究[16)]が存在するが、臨床的に構成生薬で経口投与後の適切な薬物動態プロファイリングを行った報告はない。

そこで 2011 年 4 月、TJ-54 の臨床薬物動態研究の第一歩として、ヒトに TJ-54 を投与した際に吸収される成分の探索を目的とし、「抑肝散(TJ-54)の健常人を対象とした探索薬物動態試験」を実施した[17)]。その結果、TJ-54 において薬理作用が示唆されている釣藤鉤アルカロイド成分ガイソシジンメチルエーテル[11), 18)~20)]、ヒルステイン[20, 21)]およびグリチルレチン酸[13)]が血漿中に確認された。

以上のような背景および結果を踏まえ、今回、ヒトに TJ-54 を投与した際に吸収されるガイソシジンメチルエーテル、ヒルステインおよびグリチルレチン酸の量的把握を目的とした定量的な臨床薬物動態試験を計画した。また、TJ-54 の構成生薬のひとつである甘草は、長期大量摂取により偽アルドステロン症を発症する可能性が示唆されており、この原因化合物はグリチルレチン酸やグリチルレチン酸モノグルクロナイドであるという報告[22)]がある。そこで TJ-54 服用時のグリチルレチン酸モノグルクロナイドの血漿中濃度もあわせて検討することにした。本知見は、副作用研究に対して重要なデータを提供するものと考えられる。

以上のように、今回ヒトにおける TJ-54 の薬物動態研究を行うことは、基礎薬理作用と TJ-54 成分の関係の把握、副作用解明への一助という点で有益であると思われる。

なお、本試験は「医薬品の製造販売後の調査及び試験の実施の基準に関する省令(GPSP)」、「医薬品の臨床試験の実施の基準に関する省令(GCP)」、薬事法第 14 条第 3 項および第 80 条の 2 に規定する基準ならびに本試験実施計画書を遵守して実施する。

3. 試験の目的

3.1 試験の目的

日本人健常成人を対象として、ツムラ抑肝散エキス顆粒（医療用）（TJ-54） 7.5g （1包2.5g×3包）、 5.0 g （1包2.5g×2 包）、2.5g （1包2.5g×1包）の単回経口投与時における薬物動態および安全性について検討する。

3.2 試験の種類

定量試験 （製造販売後臨床試験）

4. 対象

4.1 対象被験者

選択基準にすべて合致し、除外基準に抵触しない健常成人

4.2 目標症例数

目標完了症例数として 15 例以上

【症例数設定の根拠】

被験者数は薬物動態の評価が可能となる一般的な例数として 15 例とした。完了の定義は 5.3.1 を参照。

4.3 選択基準

1) 日本人
2) 20 歳以上～45 歳未満の者（同意取得時）
3) 肥満度（BMI）が 18.5 以上 25 未満の者
4) 性別：不問
5) 本人から文書による同意を得た者

【選択基準設定の根拠】

本試験は日本人を対象とする。また参加意思を本人が明確に判断できるものとして成人を対象としたため、年齢下限を設定し、加齢による生理機能の影響を除くため、年齢上限を設けた。また薬物の分布に影響を与える可能性があるため、BMI の範囲を日本肥満学会が定める普通体重指数とした。

4.4 除外基準

1) 薬物アレルギー（漢方薬を含む）、食物アレルギーの既往歴のある者

2) 妊婦、妊娠を希望する者、授乳している者
3) 被験薬投与前 16 週以内に、他の臨床研究に参加した者
4) 被験薬投与前 12 週以内に 400mL を超える採血・献血を行った者
5) 重篤な肝疾患、心疾患および血液疾患のうちいずれかの既往歴のある者
6) 脂肪肝の者
7) 各期の被験薬投与の 3 日前から各期の終了まで、アルコールあるいはタバコを中止できない者
8) 各期の被験薬投与の 7 日前から各期の終了まで、薬剤(たとえば向精神薬、抗真菌薬、抗高血圧薬、漢方薬等)を中止できない者
9) カンゾウ、センキュウ、トウキ、サイコ、チョウトウコウ、ソウジュツ、ブクリョウを由来とするサプリメントを被験薬投与の 3 日以内に投薬している者
10) 各期の被験薬投与の 7 日前から被験薬投与までの間に、何らかの薬剤を投薬した者
11) スクリーニング検査または投与前検査において、臨床検査の基準範囲を超え、試験責任医師等が不適当と判断した者
12) その他、試験責任医師等が不適当と判断した者
13) HCV 抗体、HBs 抗原、HIV 抗体陽性のもの

【除外基準設定の根拠】
1)～5)は被験者の安全確保のため、6)～13)は本試験の目的に影響を与える恐れのある被験者を除外するため、さらに、13)は分析者の安全のために設定した。

4.5 登録の方法

4.5.1 被験者選定

試験責任医師または試験分担医師は、以下の手順で被験者を選定する。
1) 選択基準に合致した試験参加希望者に対して、被験者同意説明文書に基づき詳細な説明をし、文書により試験参加の同意を得る。
2) 同意取得者に対してスクリーニング検査を実施し、その結果から除外基準に抵触しない被験者候補を選定する。
3) 被験者候補に対して、投与前検査を実施し、その結果から総合的に判断して被験者を選定する。

4.5.2 スクリーニング名簿/被験者登録名簿の作成

試験責任医師は、結果として同意取得の有無に関わらず、同意取得の要請ならびに情報提供を行った全ての被験者について、「スクリーニング名簿/被験者登録名簿(書式 1)」に同意説明日(同意取得の要請ならびに情報提供を行った日)および同意の取得の有無、同意取得日を記入する。
また、試験参加の同意が得られ、被験者とすることに決定した場合には、被験者識別コードおよび

性別について記入する。

4.5.3 被験者識別コードリストの作成

試験責任医師は、被験者に対して、被験薬を投与する前に被験者識別コードを設定する（被験者割付）。

また、「被験者識別コードリスト（書式 2）」を上記のスクリーニング名簿／被験者登録名簿とは別に作成し、被験者識別コード、カルテ No.および被験者名を記入する。

なお、試験責任医師が有害事象およびその他の試験関連データを報告する際には、被験者の特定は被験者識別コードにより行う。

固定コード ― 被験者番号	被験者識別コード（例）
BJ01―01、02、03、04	BJ01―01

4.5.4 被験者の割付

スクリーニング検査および投与前検査にて選定された被験者は、登録後に中央割付システムにより 3 群（A～C 群）のいずれかにランダム割付される（割付比 1:1:1）。割付表は予め作成した割付表を用いて行う。この時、以下を「被験者識別コード表（書式 2）」に記入する。

(ア) 割付可否

(イ) 割付順

(ウ) 割付群 ［A 群、B 群または C 群］

5. 試験方法

5.1 試験デザイン

ランダム化クロスオーバー試験とする。同一被験者で 4 週以上のウォッシュアウト期間をおいて実施する。

5.2 投薬の概要

被験者を登録時に A～C 群に分け、同一被験者に対して 3 種の投与量を 3 回に分けて投与する。3 回の投与時期は 1 期、2 期、3 期とし、1 期と 2 期および 2 期と 3 期は 4 週以上のウォッシュアウト期間を設ける（表 1）。

表 1 投薬の概要

	1 期	ウォッシュアウト期間	2 期	ウォッシュアウト期間	3 期
A 群	7.5g 投与	4 週以上	2.5g 投与	4 週以上	5.0g 投与
B 群	2.5g 投与	4 週以上	5.0g 投与	4 週以上	7.5g 投与
C 群	5.0g 投与	4 週以上	7.5g 投与	4 週以上	2.5g 投与

5.3　試験の完了および中止・脱落

5.3.1　完了

各期の 48 時間の採血および終了時検査を受けた場合に各期の終了とする。
3 期の終了を持って本試験の完了とする。

5.3.2　中止・脱落基準

以下の場合、試験を中止する。

1)　被験者の同意の撤回あるいは辞退申し入れ
2)　有害事象が発生し、試験責任医師により継続が困難と判断された場合
3)　被験薬を服薬できなかった場合
4)　その他、試験責任医師により継続が不適切であると判断された場合

5.3.3　中止手順

中止の場合、試験責任医師あるいは試験分担医師は、その理由、処置および経過等を症例報告書に記入する。なお、安全性上の問題により中止された場合は、被験者に対して適切な処置を実施し、被験者の同意が得られない場合を除き被験者が安全であると確認できるまで追跡して検査、調査を実施するなど、安全性確保に努める。

5.3.4　被験者の管理

臨床試験に参加している間、被験者は試験責任医師の管理下におかれる。試験責任医師は被験者が以下の内容を遵守するよう指示を行う。

1)　飲食物

各期の被験薬投与の 3 日前から各期の終了まで、カフェイン、アルコール、炭酸水および甘草含有飲食物〔原材料名に標記があるもの〕、にんにく、柑橘類（グレープフルーツ、オレンジ、みかん等）を含む飲食品（香辛料を含む）〔別紙参照〕ならびに抑肝散の構成生薬（カンゾウ、センキュウ、トウキ、サイコ、チョウトウコウ、ソウジュツ、ブクリョウ）を由来とするサプリメントの摂取を禁止する。ただし、飲料水の摂取は適宜自由とする。被験薬投与前日 20：00 以降から投与後 4 時間までは絶食とする。

2)　喫煙

各期の被験薬投与の 3 日前から各期の終了まで、禁煙とする。

3)　他剤の使用

各期、被験薬投与の 7 日前から各期の終了まで、他剤の使用を禁止する。

6. 被験薬

6.1 使用薬名および製造元

被験薬名: ツムラ抑肝散エキス顆粒(医療用)

コード番号: TJ-54

製造元: 株式会社ツムラ

6.2 成分、剤型および保管条件

成分・含量: TJ-54 7.5g 中に下記の割合の混合生薬の乾燥エキス 3.25g を含有する。

日局ソウジュツ…………4.0g 日局トウキ……3.0g

日局ブクリョウ…………4.0 g 日局サイコ……2.0g

日局センキュウ…………3.0 g 日局カンゾウ……1.5g

日局チョウトウコウ………3.0 g

添加物 : 日局ステアリン酸マグネシウム、日局乳糖水和物

剤型: 顆粒剤

包装: 1包 2.5g のヒートシール包装

保管条件: 出来るだけ湿気を避け、直射日光の当たらない涼しい所に保管すること。

6.3 投与方法および投与量

TJ-54 7.5g (1 包 2.5g×3 包)、 5.0 g (1 包 2.5g×2 包)、2.5g (1 包 2.5g×1 包)を単回経口投与する。

【投与量設定の根拠】

本試験では、以下の3つの点から 7.5g、5.0g、2.5g(公差 2.5)の 3 用量を投与量として設定した。臨床用量では通常 1 日量 7.5g を 2 ないし 3 回に分けて服用する。

① 単回投与での被験者に対する忍容性を考慮した投与量の最大用量 7.5g の設定
   - 本試験に先立って実施した探索薬物動態試験 [17] において 7.5g を単回投与したところ、被験薬に因果関係のある有害事象は生じなかった。

② TJ-54 の用法、用量からの最小用量 2.5g の設定
   本臨床試験での TJ-54 投与量の最小投与量は、TJ-54 の平均的な 1 回服用量が 2.5g であることから設定した。

③ 測定可能な濃度であるかの考察
   探索薬物動態試験 [17] の結果では、7.5g 服用での最高血漿中濃度は、ガイソシジンメチルエーテル 2.82 ng/mL、ヒルステイン 0.323 ng/mL およびグリチルレチン酸 287 ng/mL であった。現時点での定量バリデーション試験における定量下限値は、ガイソシジンメチルエーテ

ル 0.3 ng/mL、ヒルステイン 0.1ng/mL およびグリチルレチン酸 1 ng/mL が可能となるため、これら化合物は 2.5g 単回服用時でも最高血漿中濃度測定は可能と思われる。

【グリチルレチン酸モノグルクロナイドの測定について】

TJ-54 の構成生薬のひとつである甘草は、長期大量摂取により偽アルドステロン症を発症する可能性が示唆されており、動物での検討結果からグリチルレチン酸モノグルクロナイドはこの原因化合物の一つという報告もある[22)]。探索薬物動態試験[17)]の結果では、定量下限値未満であったが、本化合物の健常人における濃度データを把握することは、今後の副作用研究の際、患者データと比較する上で有用であると判断し、今回血漿中濃度測定を実施する。グリチルレチン酸モノグルクロナイドの濃度測定は、ガイソシジンメチルエーテルやヒルステインと同一の測定系の中で実施できることから、被験者に負担となる測定とはならない。

6.4 被験薬の交付

被験薬は、実施医療機関と試験依頼者の間で試験契約が締結された後、試験依頼者が実施医療機関に無償で交付する。

6.5 被験薬の保管および管理

試験薬管理者は、被験薬を適切に保管、管理する。

6.6 被験薬の回収

試験依頼者は未使用被験薬の数量等を確認した後、試験薬管理者より回収する。

7. 検査・観察項目および実施時期

7.1 検査・観察項目

7.1.1 スクリーニング検査項目

被験薬投与 7 日前に試験責任医師または試験分担医師は以下の調査・観察・検査を実施し、その結果から被験者候補を選定する。スクリーニング日の許容誤差は、±1 日とする。

被験者候補背景： 生年月日、性別、人種、現在罹患している疾患、服用している薬剤、薬物アレルギー歴、食物アレルギー歴、既往歴、アルコール摂取量、喫煙の有無

診察所見： 自覚症状、他覚所見、浮腫

理学的検査： 身長、体重、BMI、体温(腋窩)、血圧(坐位)、脈拍(坐位)

臨床検査：

1) 血液学的検査

赤血球数、白血球数、血小板数、ヘモグロビン量、ヘマトクリット値

2) 血液生化学検査

総蛋白、BUN、クレアチニン、尿酸、AST、ALT、T-Bil、ALP、γ-GTP、Alb、PT、TC、

CRP、HbA1C、K

3) 尿中一般検査

尿糖

尿妊娠検査(女性のみ)

ウイルス検査:HCV 抗体、HBs 抗原、HIV 抗体

7.1.2 投与前検査

各期の被験薬投与前日に試験責任医師または試験分担医師は以下の観察・検査を実施し、その結果から総合的に判断して被験者候補を選定する。

診察所見: 自覚症状、他覚所見、浮腫

理学的検査: 体温(腋窩)、血圧(坐位)、脈拍(坐位)

臨床検査:

1) 血液学的検査

赤血球数、白血球数、血小板数、ヘモグロビン量、ヘマトクリット値

2) 血液生化学検査

総蛋白、BUN、クレアチニン、尿酸、AST、ALT、T-Bil、ALP、γ-GTP、Alb、PT、TC、CRP、K

尿妊娠検査(女性のみ): 1 期においては、スクリーニング時に実施。

尿 pH: 随時尿を一回採取し、新鮮尿の pH および採尿時間を記録する。2 期および 3 期は、被験薬投与前日に尿 pH の検査は実施しない。

7.1.3 投与時検査

診察所見: 自覚症状、他覚所見

尿 pH: 投与後 4 時間までに随時尿を一回採取し、新鮮尿の pH および採尿時間を記録する。

7.1.4 終了時検査

各期の終了時に試験責任医師または試験分担医師は以下の観察・検査を実施する。

診察所見: 自覚症状、他覚所見、浮腫

理学的検査: 体温(腋窩)、血圧(坐位)、脈拍(坐位)

臨床検査:

1) 血液学的検査

2) 赤血球数、白血球数、血小板数、ヘモグロビン量、ヘマトクリット値

1) 血液生化学検査

総蛋白、BUN、クレアチニン、尿酸、AST、ALT、T-Bil、ALP、γ-GTP、Alb、PT、TC、CRP、K

7.1.5　血漿中 TJ-54 由来成分の探索
本試験の全採血量は 368mL [スクリーニング検査:8mL×1 回、120mL×3 期{(臨床検査:8mL×2 回、薬物動態 8mL×13 回)×3}] である。

血液採取:
1)　採血時期
投与前および投与後 15 分, 30 分, 1, 2, 3, 4, 8,10,12,14,24,48 時間(採血回数 13 回)
2)　採血方法と処理
採血時間帯に被験者の前腕部皮静脈より 1 回計 8 mL の静脈血を採取(薬物動態用 8mL 採血:抗凝固剤としてヘパリンナトリウム)し、4℃、1700G で 10 分間遠心分離し血漿を得る。得られた血漿を薬物動態用 8mL 採血の採血管から得られた血漿は 1mL ずつ分注して、分析まで-20℃以下で凍結保存する。
3)　採血時間の許容誤差ならびに誤差発生時の取り扱いについて
採血時間の記録は採血開始時間を記録する。採血時間の許容誤差は投与後 15 分, 30 分, および 1 時間採血終了までは採血時間の 10%以内とする(15 分±1.5 分, 30 分±3 分, 1 時間±6 分)。2, 3, 4 時間での採血時間の許容誤差は一律±10 分とする(2 時間±10 分,3 時間±10 分, 4 時間±10 分)。8、10、12、14、24 および 48 時間での採血時間の許容誤差は一律±30 分とする(8 時間±30 分、10 時間±30 分、12 時間±30 分、14 時間±30 分、24 時間±30 分,48 時間±30 分)。
許容誤差範囲を逸脱した場合には、その旨症例報告書に記載する。なお、得られた検体は分析に供することができる。得られた分析データの取り扱いは、別途作成する薬物動態解析計画書に従う。

[設定の根拠]
本試験では、ブランク血漿として使用する投与前採血と、投与後 15 分, 30 分, 1, 2, 3, 4, 8, 10, 12, 14, 24, 48 時間の 13 時点 8mL ずつの採血を計画した。
本試験に先立って実施した探索薬物動態試験 [17] の結果から、TJ-54 の定量的な薬物動態研究を実施するための測定可能成分としてガイソシジンメチルエーテル、ヒルステイン、グリチルレチン酸およびグリチルレチン酸モノグルクロナイドの 4 化合物が選択された。この試験結果からガイソシジンメチルエーテル、ヒルステインは最高血漿中濃度到達時間(tmax)が投与 2 時間以内であることが予想された。またグリチルレチン酸は tmax が約 13 時間であることが知られている。そこで、本試験では採血時間を計画のように設定した。
定量する各成分の予想される tmax を考慮し、各成分の薬物動態学的特性を確認できる採血ポイントとして、各採血時間を設定した。さらに、グリチルレチン酸の半減期が 7.4±2.7 時間であることから、3～5 半減期まで確認できる期間として最終採血時間を 48 時間に設定した。日本国内の薬物動態試験ガイドライン(血中代謝物の濃度推移の確認や薬物動態パラメーターの算出

を実施する試験)では、測定成分の半減期の 3 倍までの測定期間の設定が求められている。米国 FDA は、測定成分の 5 半減期を設定するよう求めている。米国 FDA からの要求にも対応可能な最終採血時間として、今回の試験では、48 時間を設定した。

採血量は分析必要量から設定した。薬物動態の 1 回の分析に使用する血漿量は、再測定の可能性も考慮し 3mL 程度と算出した。従って、採取する血液量を 8mL(抗凝固剤としてヘパリンナトリウム)と計画した。

7.1.6 安全性項目

有害事象

7.2. 実施スケジュール

各検査・観察項目は表 2 の試験実施スケジュールに従って行う。

表 2 試験実施スケジュール

	Day-7	1期		4 週以上	2 期		4 週以上	3 期	
		Day-1	Day0～Day+1		Day-1	Day0～Day+1		Day-1	Day0～Day+1
	スクリーニング	投与前日	投与、採血 24 時間まで)		投与前日	投与、採血(24 時間まで)		投与前日	投与、採血(24 時間まで)
同意取得	○			ウォッシュアウト期間			ウォッシュアウト期間		
被験者登録		○							
割付			○						
被験者背景	○								
身長・体重・BMI	○								
血圧・脈拍・体温	○	○	○		○	○		○	○
診察	○	○	○		○	○		○	○
尿妊娠検査	○				○			○	
臨床検査	○	○	○		○	○		○	○
TJ-43 服用			○			○			○
A 群			7.5 g			2.5 g			5 g
B 群			2.5 g			5 g			7.5 g
C 群			5 g			7.5 g			2.5 g
薬物動態			○			○			○
有害事象		←							→

各検査・観察項目は表 3 の試験実施スケジュールに従って行う（タイムスケジュール例を記載）。
原則として、その翌日に入院していただき抑肝散を服用し、4 時間後までの採尿および 24 時間後までの診察、検査、採血にご協力いただき一旦退院、48 時間採血に際して再度来院とする。

表 3　試験実施スケジュール （各期）注 1

	来院		入院														来院
	Day -7	Day -1	Day 0												Day +1	Day +2
	スクリーニング	投与前日	投与直前	0	15 min	30 min	1 hr	2 hr	3 hr	4 hr	8 hr	10 hr	12 hr	14 hr	24 hr	48 hr
			7:30	8:00	8:15	8:30	9:00	10:00	11:00	12:00	16:00	18:00	20:00	22:00	8:00	8:00
同意取得	○															
被験者登録		○														
割付			○													
被験者背景	○															
身長・体重・BMI	○															
血圧・脈拍・体温	○	○														○
診察	○	○	○													○
尿妊娠検査注 2	○															
臨床検査注 3（採血量 mL）	○ 8mL	○ 8mL														○ 8mL
TJ-54 服用				○												
食事注 4		○								○		○			○	○
薬物動態注 3（採血量 mL）			○ 8mL		○ 8mL	○ 8mL	○ 8mL	○ 8mL	○ 8mL	○ 8mL	○ 8mL	○ 8mL	○ 8mL	○ 8mL	○ 8mL	○ 8mL
尿pH 注 5		○	←						→							
有害事象		←														→

注1：上記は 1 期のスケジュールであり、2 期および 3 期では抑肝散服用 7 日前のスクリーニングを

実施せず、服用前日の検査から実施する。

注2: 女性のみ実施する。1 期においては、スクリーニング時に実施する。2 期および 3 期においては、TJ-54 服用前日に実施する。

注3: 本試験の全採血量は 368mL [スクリーニング検査:8mL×1 回、120mL×3 期{(臨床検査: 8mL×2 回、薬物動態 8mL×13 回)×3}] である。なお、4 時間後、12 時間後、24 時間後および 48 時間後の採血は、食事前に実施する。

注4: 被験薬投与の各期の 3 日前から試験終了まで、甘草、カフェイン、アルコール、炭酸水、にんにく、柑橘類(グレープフルーツ、オレンジ、みかん等)を含む飲食品(香辛料を含む)〔別紙参照〕ならびに抑肝散の構成生薬(カンゾウ、センキュウ、トウキ、サイコ、チョウトウコウ、ソウジュツ、ブクリョウ)を由来とするサプリメントの摂取を禁止する。投与前日の食事は 20:00 までとし、前日 20:00 以降から投与 4 時間後採血終了後までは絶食とする。飲料水の摂取は適宜自由とする。

注5: 服用前日および服用後 4 時間までに随時尿を一回ずつ採取し、新鮮尿の pH および採尿時間を記録する。2 期および 3 期は、服用前日に尿 pH の検査は実施しない。

8. 評価の基準および解析

8.1 対象症例

8.1.1 薬物動態に関する解析症例

登録症例のうち、選択基準に合致し、除外基準に抵触しない症例であり、被験薬を服用し、薬物動態用の採血がなされた被験者

8.1.2 安全性に関する解析症例

被験薬を服用した症例

8.2 評価項目

8.2.1 薬物動態

採取された血液を使用し、定量バリデーション試験を行った 4 化合物(ガイソシジンメチルエーテル、ヒルステイン、グリチルレチン酸およびグリチルレチン酸モノグルクロナイド)の血漿中濃度及び血漿中濃度から求めた最高血漿中濃度(C_{max})、最高血漿中濃度到達時間(t_{max})、薬物血中濃度－時間曲線下面積(AUC)などの薬物動態学的パラメータ算出などの解析を行い、薬物動態を評価する。

これらの 4 化合物の血漿中濃度分析方法の詳細は別途作成する試験計画書に従う。分析は積水メディカル株式会社にて実施する。ただし、必要に応じて探索的な代謝物検索等を含めた追加分析を実施する。

薬物動態解析は、別途薬物動態解析計画書に詳細を記載する。薬物動態解析は薬物動態解析計画書に従って、ツムラ研究所で実施する。採血時間の許容誤差範囲を逸脱した場合のデータの

取り扱いについては薬物動態解析計画書の定めに従う。また、血漿中濃度測定値を含む得られた各種薬物動態に関するデータは後日別途解析に使用する場合がある。
薬物動態解析を行う４化合物のTJ-54 薬剤ロット中の含量は、使用するTJ-54 薬剤ロットを血漿中濃度分析の際に、積水メディカル株式会社で分析することにより求める。

8.2.2　安全性評価

診察所見、理学的検査所見、臨床検査所見を総合し、安全性を評価する。なお、診察所見、理学的検査所見、臨床検査所見は原則として、投与前検査における値を初期値とし、投与後の変化について勘案する。
［異常変動有無の判定］
- 試験責任医師は、試験薬投与前後の値を比較して異常変動を判定する。
- 異常変動ありと判定した項目は有害事象として取り扱い有害事象欄に記入する。

8.2.3　症例固定

薬物動態に関する解析対象集団ならびに安全性に関する解析対象集団を確定するため、試験依頼者はツムラ-GCP/SOP/11 に従い症例固定を行う。

8.3　統計解析計画

解析に用いる評価項目の特定、分類、およびこれらの項目の解析方法ならびにその実施時期については、「統計解析計画書（別紙 2）」に従う。また、統計解析は、統計解析方法指示書を作成した上で実施する。

9.　有害事象

9.1　定義

9.1.1　有害事象

有害事象とは、各期の試験中、あらゆる好ましくないあるいは意図しない徴候（臨床検査値の異常を含む）、症状または病気のことであり、被験薬との因果関係の有無は問わない。試験中に発現した疾患、試験の開始以前から認められていた疾患の悪化も、有害事象と判断する。

9.1.2　重篤な有害事象

有害事象のうち、以下にいずれかに該当するものを指す。

（1） 死に至るもの
（2） 生命を脅かすもの
（3） 治療のための入院もしくは入院の延長が必要となるもの
（4） 永続的または顕著な障害・機能不全に陥るもの

(5) 先天異常を来たすもの
(6) その他の重大な医学事象

9.1.3　副作用
有害事象のうち被験薬との因果関係が否定できないもの（9.1.4 被験薬との因果関係が 1～3 のもの）を指す。

9.1.4　被験薬との因果関係
以下の判定基準を用いて 4 段階で判定する。

【因果関係の判定基準】
1） 明らかに関連あり
被験薬の投与と有害事象の発現に時間的に明白な相関関係（投与中止後の経過も含む）があり、確かな理由およびそれを裏付ける十分な証拠がある場合。
2） おそらく関連あり
被験薬の投与と有害事象の発現に時間的に明白な相関関係（投与中止後の経過も含む）があり、本試験の当該薬剤以外の要因がほぼ除外される場合。
3） おそらく関連なし
原疾患、合併症、併用薬剤、併用療法など他の要因が推定されるが、被験薬の投与と有害事象の発現に時間的に明白な相関関係（投与中止後の経過も含む）があり、本試験の当該薬剤による可能性も除外できない場合。
4） 関連なし
被験薬の投与と有害事象の発現時間に相関関係がなく、原疾患、合併症、併用薬剤、併用療法など他の要因によると考えられるなど、因果関係を否定する十分な根拠がある場合。

9.1.5　有害事象の重症度
1） 軽度：
通常、一過性で、被験者の日常生活を損なわず、治療を要しない程度（正常な活動が可能である）
2） 中等度：
被験者の日常生活に多少の支障をきたし、十分な不快感を与え、治療を要する程度（活動に不快感を伴う）
3） 高度：
被験者の日常生活の遂行に大きな支障があり、治療を要する程度（正常な活動が困難である）

9.2　有害事象のモニタリング

問診および観察により有害事象の有無を調査し、有害事象が認められた場合には、追跡調査を実施するとともに、以下の項目について症例報告書に記入する。

ｉ）有害事象名
ii）発現日
iii）重篤度（1.重篤でない　2.重篤）
iv）重篤理由およびその他の重大な医学事象にあってはその理由
ｖ）重症度（1.軽度　2.中等度　3.高度）
vi）処置（治療の有無および治療内容）
vii）転帰（1.回復(消失)　2.軽快　3.未回復(不変)　4.後遺症　5.不明）
viii）転帰確認日
ix）因果関係（1.明らかに関連あり 2.おそらく関連あり 3.おそらく関連なし 4.関連なし）

試験責任医師または試験分担医師が臨床上重要と判断した臨床検査値の異常変動については、その根拠とともに症例報告書に記入する。
有害事象と被験薬との因果関係がない場合は、その判定の根拠を症例報告書に記入する。

9.2.1　発生時の対応

被験薬との因果関係の有無にかかわらず、9.1.2 項において重篤と判断された有害事象が発生した場合、試験責任医師は適切な処置をとるとともに、速やか（24 時間以内）にその所属する実施医療機関の長または治験審査委員会および試験依頼者に口頭、電話または FAX で連絡し、数日中に「重篤な有害事象に関する報告書（書式 3）」を用い文書にて報告する。また、試験責任医師および試験分担医師は、試験依頼者、実施医療機関の長および治験審査委員会の要求に応じて追加情報を提供する。
実施医療機関の長は当該実施医療機関において試験を継続して行うことの適否について治験審査委員会の意見を聞く。
試験依頼者は、ツムラ-GCP/SOP およびツムラ-GPSP/SOP、ならびに薬事法第 77 条の 4 の 2 および同施行規則第 253 条の規定に従って、使用上の注意から予測できる重篤な副作用および使用上の注意から予測できない重篤または重篤でない副作用と疑われる症例に関しては、規制当局へ速やかに報告する。

9.3　新たな情報の提供

添付文書の改訂等の被験薬の安全性等に関する新たな情報を得た場合、試験依頼者は速やかに実施医療機関の長または治験審査委員会および試験責任医師に文書で報告し、必要な措置を講ずる。

9.4　予測される副作用

添付資料「ツムラ抑肝散エキス顆粒（医療用）添付文書」参照

10. 倫理的事項

10.1 倫理原則の遵守

本試験は、ヘルシンキ宣言の精神を遵守し、かつ本試験実施計画書、第 80 条の 2 に規定される基準ならびに平成 9 年 4 月 1 日より施行された「医薬品の臨床試験の実施に関する基準（GCP）」（厚生省令第 28 号）および関連省令・通知書を遵守して実施する。

なお、必要な場合には、随時試験実施計画書等の改訂を行うこととする。

10.2 治験審査委員会

試験実施に先立ち、実施医療機関の治験審査委員会において、試験実施計画書、症例報告書様式、被験者への同意説明文書の記載内容等、および当該機関における試験実施の適否に関して審査を行うこととする。

10.3 インフォームドコンセント

10.3.1 同意取得の時期と方法

試験責任医師または試験分担医師は、対象となる被験者に対して、被験薬投与 1 週間前に、作成した同意説明文書に基づいて、10.3.2 項の内容を十分に説明し、被験者が内容をよく理解したことを確認した上で、本試験への参加について被験者の自由意思による同意を、文書にて得るものとする。同意文書には、説明を行った試験責任医師または試験分担医師および被験者が記名捺印または署名し、各自日付を記入する。また、同意説明日および同意取得日を症例報告書に記載するとともに、同意書を実施医療機関の定める部署へ提出するか、または診療録に貼付し、同意書の写しを含む同意説明文書を被験者に提供する。

試験参加の継続に関して、被験者の意思に影響を与える可能性のある情報が得られた場合には、試験責任医師または試験分担医師は当該情報を速やかに被験者に伝え、試験に継続して参加するか否かについて被験者の意思を確認し、その旨を日付とともに文書に記載する。

10.3.2 同意説明文書による説明事項

試験の内容などの下記事項について、同意説明文書を用いて被験者に説明する。

(1)　本試験が研究を伴うこと。

(2)　試験の目的（必要とされる食事制限の内容についても知らせること）。

(3)　試験責任医師の職名および連絡先。

(4)　試験の方法（試験の研究的側面、被験者の選択基準）。

(5)　予期される臨床上の利益および危険性または不便（被験者にとって予期される利益

がない場合には、被験者にその旨を知らせること）。

(6)　被験者の試験への参加予定期間および時間。

(7)　試験への参加は被験者の自由意思によるものであり、被験者は、試験への参加を随時拒否または撤回することができること。また、拒否・撤回によって被験者が不利な扱いを受けたり、試験に参加しない場合に受けるべき利益を失うことはないこと。

(8)　モニター、監査担当者、治験審査委員会および規制当局が医療に係る原資料を閲覧できること。その際、被験者の秘密は保全されること。また、同意文書に被験者が記名捺印または署名することによって閲覧を認めたことになること。

(9)　試験の結果が公表される場合であっても、被験者の秘密は保全されること。

（10）　被験者が試験および被験者の権利に関してさらに情報の入手を希望する場合または試験に関連する健康被害が生じた場合に照会すべきまたは連絡をとるべき実施医療機関の相談窓口。

（11）　試験に関連する健康被害が発生した場合に被験者が受けることのできる補償および治療。

（12）　試験に参加する予定の被験者数

（13）　試験への参加の継続について被験者の意思に影響を与える可能性のある情報が得られた場合には速やかに被験者に伝えること。

（14）　試験への参加を中止させる場合の条件または理由。

（15）　被験者が費用負担する必要がある場合にはその内容。

（16）　被験者に金銭等が支払われる場合にはその内容（支払額算定の取り決め等）。

（17）　被験者が守るべき事項。

10.3.3 同意説明文書の承認と改訂

試験責任医師は、本試験の実施に先立ち、予め治験審査委員会の承認が得られた同意説明文書を作成する。

また、本試験の当該薬剤に関連する安全性等に関する新しい情報を得た場合、速やかに医療機関の長または治験審査委員会に文書で報告し、必要な措置を講ずる。その際に、医療機関の長または治験審査委員会のいずれかが同意説明文書の改訂が必要と判断した場合には、速やかに改訂を行うこととする。

試験責任医師または試験分担医師は改訂された同意説明文書を用いて改めて説明し、試験への参加の継続について被験者から自由意思による同意を文書により得なければならない。

試験責任医師または試験分担医師は、同意を得た年月日を症例報告書に記入するとともに、新たに記名捺印または署名と日付を記入した同意書の写しと同意説明文書を被験者に渡さなければならない。

10.4 個人情報の保護

症例報告書等における被験者の記載は、被験者識別コードで特定するなど第三者が直接その被験者を識別できないよう十分に配慮する。すなわち、被験者の氏名やイニシャルは使用せず、登録症例の特定や照会は、登録時に発行される被験者識別コードを用いて行う。試験の結果を学会等で公表する場合には被験者を特定できないように行う。

10.5 金銭の支払い

被験者の不便性の解消を目的として、実施医療機関の規定あるいは実施医療機関との協議に基づき、基準に従って交通費等の協力費を支払うものとする。なお、支払い方法については、別途実施医療機関と協議する。

10.6 健康被害補償および保険

本試験の実施に起因して被験者に何らかの健康被害が生じた場合には、治療その他必要な処置をとるものとする。また、試験依頼者は、被験者の健康被害の補償を履行するための保険に加入する等の措置を講じる。なお、健康被害が生じた際、試験依頼者は試験責任医師と協議の結果、それが補償の対象となる旨判断される場合および実施医療機関または被験者より補償の申し入れがなされた場合には、直ちに試験実施責任者、総括責任者と協議の上、ツムラ-GCP/SOP に従った措置をとるものとする。その場合、医療費は全額(但し、健康保険等からの給付を受けられる時には被験者の自己負担額)を、医療手当は治療のために入院を要する程度以上の健康被害に対して医薬品副作用被害救済制度の給付に準じた額を、補償金は労働者災害補償保険に準じた額を治験依頼者が負担する。

10.7 試験の終了または中止および中断

10.7.1 試験の終了

試験責任医師は試験終了後、速やかに実施医療機関の長に以下の事項を記載した試験終了報告書を提出する。その後、実施医療機関の長は試験依頼者へ試験終了通知書を提出する。

(1) 試験終了報告書の提出年月日
(2) 試験依頼者の名称、所在地
(3) 試験課題名
(4) 試験責任医師および試験分担医師の氏名(全員を記載)
(5) 試験実施期間
(6) 実施症例数
(7) 試験成績の概要
(8) GCP の遵守状況

10.7.2 試験全体の中止または中断の基準

試験実施中に以下のような状況となった場合には試験全体を中止または中断する。

(1) 重篤な有害事象が発生し、試験継続が困難と判断される場合
(2) 有害事象が高頻度に発生し、試験継続が困難と判断される場合
(3) 試験以外の新たな基礎試験成績において、被験者に重大な影響を及ぼすと推測される成績が得られた場合
(4) その他試験依頼者が試験全体を中止または中断すべきとの判断を行った場合

10.7.3 試験中止または中断の手続き

試験の途中で上記基準により試験全体を中止あるいは中断せざるを得ない理由が生じた場合、試験依頼者は速やかに試験の中止・中断およびその理由を試験責任医師および実施医療機関の長または治験審査委員会に文書で報告する。

また、試験責任医師は、被験者に速やかにその旨を通知するとともに、必要な措置を講じるとともに、被験者の試験に関するデータを速やかに症例報告書に記入し、試験依頼者に提出する。

11. 試験実施計画書の遵守および逸脱または変更ならびに改訂

11.1 試験実施計画書の遵守

本試験は、試験責任医師と試験依頼者の合意のもとに本試験実施計画書を遵守して実施する。

11.2 試験実施計画書の逸脱または変更

試験実施計画書は原則として逸脱または変更はできないが、医療上やむを得ない事情や治験審査委員会の意見に基づく実施医療機関の長の意見があった場合には、この限りではない。

試験責任医師または試験分担医師は、試験実施計画書から逸脱した行為を全て記録し、その理由等を説明した記録を試験依頼者に提出し、その写しを保存する。

試験責任医師または試験分担医師は被験者の緊急の危険を回避する等、医療上やむを得ない事情のために、試験依頼者との事前の文書による合意および治験審査委員会の事前の承認なしに試験実施計画書からの逸脱または変更を行うことができる。

試験責任医師は、試験の実施に重大な影響を与え、または被験者の危険を増大させるような試験のあらゆる変更について、試験依頼者、実施医療機関の長および治験審査委員会に速やかに報告書を提出する。

11.3 実施計画書の改訂

試験開始後、試験実施計画書の変更を余儀なくされた場合、試験依頼者は変更内容を実施医療機関の長および試験責任医師に連絡する。実施医療機関の長は、変更の程度に応じて治験審査委員会に対し報告または審査依頼を行い、その結果を試験責任医師に指示する。試験依頼者およ

び試験責任医師がこの変更について合意した旨を証するため、試験責任医師は、試験依頼者とともに変更された試験実施計画書またはそれに代わる文書に記名捺印または署名し、日付を記入するものとする。

12. データの収集および保存

12.1 症例報告書の作成

試験を担当した試験責任医師または試験分担医師は、全ての観察、検査が終了後、速やかに症例報告書を作成し、内容を確認後、症例報告書の表紙の医師名欄に記名捺印または署名する。
また、試験分担医師が作成した症例報告書については、試験依頼者に提出される前に必ず試験責任医師がその内容を点検し、問題がないことを確認の上、試験責任医師欄に記名捺印または署名する。
なお、症例報告書に用いる印鑑は、予め作成した「署名・印影一覧表（書式 4）」と同一のものとする。

12.2 記録の保存

被験者の同意に関する記録、症例報告書作成の基礎となった記録（診療録、検査データ等）、治験審査委員会の審議資料および記録、試験の依頼・契約に係る書類および試験薬管理表等、実施医療機関において保存すべき文書または記録については、実施医療機関の長の定めた記録保存責任者が本試験の中止若しくは終了の後 3 年間保存する。但し、移転その他の理由により移動させる場合は、移動先を試験依頼者に通知する。

13. 原資料の直接閲覧

本試験の試験責任医師および本試験を受け入れる実施医療機関は、治験審査委員会、試験に関連するモニタリング担当者、試験依頼者の監査担当者ならびに治験審査委員会および規制当局の調査の際に、原資料等すべての試験関連記録を直接閲覧に供さなければならない。

13.1 原資料の特定

試験責任医師は、試験開始前に、症例報告書に記載されるデータに関する原資料、保存場所および保存責任者を特定して、「原資料一覧表（書式 5）」を作成し、試験依頼者との間で合意しておく。
但し、症例報告書中の各コメント欄、臨床検査値の異常変動の有無、有害事象の有無、副作用の有無、因果関係の判定については症例報告書を原資料とする。

13.2 直接閲覧の方法

原資料等の直接閲覧の詳細な実施方法に関しては、試験開始前に、試験依頼者と試験責任医師または実施医療機関との間で実施要領を協議、決定する。(「直接閲覧実施要領」参照)

13.3 閲覧による照合・確認結果の評価および対応

症例報告書の記載内容と原資料との間に何らかの矛盾がある場合には、試験責任医師はその理由を説明する記録を作成して試験依頼者に提出し、その写しを保存する。

また、試験実施計画書からの逸脱・不遵守が認められた場合、試験依頼者は、これらの逸脱・不遵守事項を記録し、試験責任医師に速やかに報告するとともに、試験責任医師に試験実施計画書を遵守し適正に実施するよう再度説明して要請する。

14. 試験の品質管理および保証

14.1 品質管理

試験実施部門、統計解析部門および試験薬管理責任者の活動等、試験関連の活動の質が求められる事項を充足しているか否かを検証することを目的とし、試験依頼者の試験関連部門がツムラ-GCP/SOP に従い品質管理を行う。

14.2 品質保証

試験が試験実施計画書、ツムラ-GCP/SOP、ツムラ-GCP および医薬品の臨床試験の実施に関する基準(GCP)を遵守して行われているか否かを評価することを目的とし、試験依頼者の GCP 監査部門がツムラ-GCP 監査手順書に基づいた計画に従い GCP 監査を行う。なお、試験実施医療機関および試験の実施に関わる施設に対する GCP 監査についても行う。

15. 公表に関する取り決め

本試験の結果については、試験依頼者の同意なしに学会発表および医学雑誌に投稿してはならない。

16. 試験期間

2012 年 5 月～2012 年 12 月

17. 試験実施体制

17.1 試験依頼者

17.1.1 試験依頼者

株式会社ツムラ　育薬企画部

〒107-8521　東京都港区赤坂二丁目 17 番 11 号

【連絡先】

TEL:03-6361-7185　FAX:03-5574-6664

17.1.2 試験依頼者の試験組織

試験依頼者の組織は、「試験依頼者の試験組織(別紙 1)」に示す。

17.1.3 メディカルアドバイザー

東京医科大学病院　老年病科　教授　羽生　春夫

〒160-0023　東京都新宿区西新宿6-7-1

TEL: 03-3342-6111　FAX:03-3342-2305

【メディカルアドバイザー(医学専門家)の役割】

医学専門家として、本試験実施全般に関し医学的な見地より助言を行う者。特に有害事象については、試験実施部門より報告を受けその内容の確認を行う。

17.2 血漿および尿中 TJ-54 由来成分の分析施設

積水メディカル株式会社　薬物動態研究所

〒319-1182　茨城県那珂郡東海村村松 2117

TEL:029-282-0234(代)　FAX :029-282-0109

17.3 試験実施医療機関および試験責任医師

試験実施医療機関:高知大学医学部附属病院

〒783-8505　高知県南国市岡豊町小蓮

TEL:088-866-5811

試験責任医師:高知大学医学部附属病院　外科(一)　教授　花﨑　和弘

18. 参考文献

1) Shinno H. et al. Effect of Yi-Gan San on psychiatric symptoms and sleep structure at patients with behavioral and psychological symptoms of dementia. Pro Neuro-Pharm Biol Psychi. 2009, 32, p.881-885.

2) Iwasaki K. et al. A randomized, observer-blind, controlled trial of the traditional Chinese medicine Yi-Gan San for improvement of behavioral and psychological symptoms and activities of daily living in dementia patients. J Clin Psychiatry. 2005, 66, p.248-252.

3) Iwasaki K. et al. Effects of the traditional Chinese herbal medicine Yi-Gan San for cholinesterase inhibitor-resistant visual hallucinations and neuropsychiatric symptoms in patients with dementia with Lewy bodies. J Clin Psychiatry. 2005, 66, p.1612-1613.

4) Mizukami K. et.al. A randomized crossover study of a traditional Japanese medicine (Kampo) "Yokukansan" in the treatment of the behavioral and psychological symptoms of dementia. Int. J. Neuropsychopharm. 2009, 12, p.91-99.

5) Monji A. et.al. Effect of yokukansan on the behavioral and psychological symptoms of dementia in elderly patients with Alzheimer's disease. Pro Neuro-Pharm Biol Psychi. 2009, 33, p.308-311.

6) Ikarashi Y. et al. Effects of yokukansan, a traditional Japanese medicine, on memory disturbance and behavioral and psychological symptoms of dementia in thiamine-deficient rats. Biol Pharm Bull, 2009, 32, p.1701-1709.

7) Tabuchi M. et al. Ameliorative effects of yokukansan, a traditional Japanese medicine, on learning and non-cognitive disturbances in the Tg2576 mouse model of Alzheimer's disease. J Ethnopharmacol. 2009, 122, p.157-162.

8) Sekiguchi K. et al. Effects of yokukansan, a traditional Japanese medicine, on aggressiveness induced by intracerebroventricular injection of amyloid β protein into mice. Phytother Res. 2009, 23, p.1175-1181.

9) Kanno H. et al. Effect of yokukansan, a traditional Japanese medicine, on social and aggressive behaviour of para-chloroamphetamine-injected rats. J Pharm Pharmacol. 2009, 61, p.1249-1256.

10) Egashira N. et al. Repeated administration of Yokukansan inhibits DOI-induced head-twitch response and decreases expression of 5-hydroxytryptamine (5-HT) 2A receptors in the prefrontal cortex. Prog Neuropsychopharmacol Biol Psychiatry. 2008, 32, p.1516-1520.

11) Terawaki K. et al. Partial agonistic effect of yokukansan on human recombinant serotonin 1A receptors expressed in the membranes of Chinese hamster ovary cells. J Ethnopharmacol. 2010, 127, p.306-312.

12) Kawakami Z. et al. Neuroprotective effects of yokukansan, a traditional Japanese medicine, on glutamate-mediated excitotoxicity in cultured cells. Neurosci. 2009, 159, p.1397. -1407.

13) Kawakami Z. et al. Glycyrrhizin and its metabolite 18β-glycyrrhetinic acid in glycyrrhiza, a constituent herb of yokukansan, ameliorate thiamine deficiency-induced dysfunction of glutamate transport in cultured rat cortical astrocytes. Eur J Pharmacol. 2010, 626, p.154-158.

14) Takeda A. et al. Suppressive effect of Yokukansan on excessive release of glutamate and aspartate in the hippocampus of zinc-deficient rats. Nut Neurosci. 2008, 11, p.41-46.

15) Takeda A. et al. Attenuation of abnormal glutamate release in zinc deficiency by zinc and yokukansan. Neurochem Int. 2008, 53, p.230-235.

16) ツムラ社内資料

17) 抑肝散(TJ-54)の健常人を対象とした探索薬物動態試験

18) Kanatani H, et al. The active principles of the branchlet and hook of Uncaria sinensis Oliv. examined with a 5-hydroxytryptamine receptor binding assay. J Pharm Pharmacol. 1985, 37(6), p.401-404.

19) Sakakibara I, et al. Effect on locomotion of indole alkaloids from the hooks of uncaria plants. Phytomedicine. 1999, 6(3), p.163-168.

20) Shimada Y,et al. Evaluation of the protective effects of alkaloids isolated from the hooks and stems of Uncaria sinensis on glutamate-induced neuronal death in cultured cerebellar granule cells from rats. J Pharm Pharmacol. 1999, 51(6), p.715-722.

21) 矢野真吾 特集 釣藤 釣藤の薬理 1987, 8(3), p.47-51.

22) Makino T. et al. Down-regulation of a hepatic transporter Mrp2 is involved in alteration of pharmacokinetics of glycyrrhizin and its metabolites in a rat model of chronic liver injury. 2008 Drug Metab. Dispos. 36(7) p1438-1443

19. 統計解析責任者及びメディカルアドバイザー（医学専門家）の確認欄
【Ver. 1.0　2011 年 12 月 27 日作成】

統計解析責任者　　　　　　　　　　　　　　　　2012 年　　月　　日

メディカルアドバイザー　　　　　　　　　　　　2012 年　　月　　日

20. 本試験実施計画書遵守の合意

合 意 書

「抑肝散（TJ-54）製造販売後臨床試験（TJ-54-4-3）」に関する試験実施計画書（Ver.1.0）および症例報告書（Ver.1.0）の内容について協議し、その内容に合意するとともに、本試験実施計画書を遵守することに合意する。

試験責任医師　　2012 年　　月　　日

医療機関名　高知大学医学部附属病院

所属・職名　外科（一）・教授

氏名　花﨑 和弘　印

試験実施責任者　　2012 年　　月　　日

株式会社 ツムラ

育薬企画部長

前村 和也　印
