# Supplementary material for: Pharmacokinetics of Active Components of Yokukansan, a Traditional Japanese Herbal Medicine after a Single Oral Administration to Healthy Japanese Volunteers: A Cross-Over, Randomized Study
Source: PLoS One. 2015 Jul 7;10(7):e0131165. doi: 10.1371/journal.pone.0131165 (PMC4495062; doi:10.1371/journal.pone.0131165)
Supplement: S2 Protocol — (DOC) [file pone.0131165.s003.doc]

| TJ-54-4-3 |
| --- |
| Ver. 1.0 |
| Prepared on December 27, 2011 |

Pharmacokinetic Study of Single Oral Administration of Yokukansan (YKS:TJ-54) in Healthy Volunteers

Post-Marketing Clinical Trial Protocol

<<English Translation from Japanese Original>>

Ver. 1.0

Prepared on December 27, 2011

| Sponsor | Tsumura & Co. Kampo Research Planning Department |
| --- | --- |
|  | 2-17-11 Akasaka Minato-ku, Tokyo, 107-8521, Japan  TEL. +813-6361-7185(direct) |

[1 STUDY SYNOPSIS 1](#__RefHeading___Toc402443217)

[1.1 TITLE 1](#__RefHeading___Toc402443218)

[1.2 STUDY OBJECTIVE 1](#__RefHeading___Toc402443219)

[1.3 ENDPOINTS 1](#__RefHeading___Toc402443220)

[1.4 STUDY POPULATION 1](#__RefHeading___Toc402443221)

[1.4.1 Subjects 1](#__RefHeading___Toc402443222)

[1.4.2 Target Sample Size to Complete Study 1](#__RefHeading___Toc402443223)

[1.4.3 Inclusion Criteria 1](#__RefHeading___Toc402443224)

[1.4.4 Exclusion Criteria 1](#__RefHeading___Toc402443225)

[1.5 STUDY DESIGN 2](#__RefHeading___Toc402443226)

[1.6 DOSAGE AND ADMINISTRATION 2](#__RefHeading___Toc402443227)

[1.7 SCHEDULE OF TIME AND EVENTS 2](#__RefHeading___Toc402443228)

[1.8 OTHER FACTORS FOR CONTROL 3](#__RefHeading___Toc402443229)

[1.9 STUDY PERIOD 3](#__RefHeading___Toc402443230)

[1.10 INSTITUTION 3](#__RefHeading___Toc402443231)

[1.11 SPONSOR 4](#__RefHeading___Toc402443232)

[2 BACKGROUND 4](#__RefHeading___Toc402443233)

[3 STUDY OBJECTIVE 5](#__RefHeading___Toc402443234)

[3.1 Study Objective 5](#__RefHeading___Toc402443235)

[3.2 TYPE OF STUDY 5](#__RefHeading___Toc402443236)

[4 STUDY POPULATION 5](#__RefHeading___Toc402443237)

[4.1 Subjects 5](#__RefHeading___Toc402443238)

[4.2 Target Sample Size 5](#__RefHeading___Toc402443239)

[4.3 Inclusion Criteria 6](#__RefHeading___Toc402443240)

[4.4 Exclusion Criteria 6](#__RefHeading___Toc402443241)

[5 STUDY METHOD 7](#__RefHeading___Toc402443242)

[5.1 STUDY DESIGN 7](#__RefHeading___Toc402443243)

[5.2 DOSAGE AND ADMINISTRATION 7](#__RefHeading___Toc402443244)

[5.3 STUDY COMPLETION; SUBJECT DISCONTINUATION, WITHDRAWAL OR REPLACEMENT 8](#__RefHeading___Toc402443245)

[5.3.1 Study Completion 8](#__RefHeading___Toc402443246)

[5.3.2 Subject Discontinuation or Withdrawal 8](#__RefHeading___Toc402443247)

[5.3.3 Discontinuation Procedure 8](#__RefHeading___Toc402443248)

[5.3.4 MANAGEMENT OF SUBJECTS 8](#__RefHeading___Toc402443249)

[6 CLINICAL TRIAL MATERIAL (CTM) 9](#__RefHeading___Toc402443250)

[6.1 PROPRIETARY NAME AND MANUFACTURER 9](#__RefHeading___Toc402443251)

[6.2 CONSTITUENTS, DOSAGE FORM, STORAGE 9](#__RefHeading___Toc402443252)

[6.3 DOSAGE AND ADMINISTRATION 10](#__RefHeading___Toc402443253)

[6.4 SUPPLY AND DELIVERY 10](#__RefHeading___Toc402443254)

[6.5 STORAGE AND HANDLING 10](#__RefHeading___Toc402443255)

[6.6 RETRIEVAL 10](#__RefHeading___Toc402443256)

[7 MEASUREMENTS AND EVALUATIONS 10](#__RefHeading___Toc402443257)

[7.1 INSPECTION AND OBSERVATION ITEMS 10](#__RefHeading___Toc402443258)

[7.1.1 Screening Items 10](#__RefHeading___Toc402443259)

[7.1.2 Predose Tests 11](#__RefHeading___Toc402443260)

[7.1.3 Test at the Administration 12](#__RefHeading___Toc402443261)

[7.1.4 Tests at the End of Study 12](#__RefHeading___Toc402443262)

[7.1.5 Search of TJ-54 Constituents in the Plasma 12](#__RefHeading___Toc402443263)

[7.1.6 Safety Parameters 13](#__RefHeading___Toc402443264)

[7.2 SCHEDULE OF TIME AND EVENTS 13](#__RefHeading___Toc402443265)

[8 CRITERIA AND ANALYSIS FOR EVALUATION 16](#__RefHeading___Toc402443266)

[8.1 TARGET POPULATION 16](#__RefHeading___Toc402443267)

[8.1.1 Target Population for Pharmacokinetic Analysis 16](#__RefHeading___Toc402443268)

[8.1.2 Target Population for Safety Analysis 16](#__RefHeading___Toc402443269)

[8.2 ENDPOINTS 16](#__RefHeading___Toc402443270)

[8.2.1 Pharmacokinetics 16](#__RefHeading___Toc402443271)

[8.2.2 Safety assessment 17](#__RefHeading___Toc402443272)

[8.2.3 Handling of Subjects 17](#__RefHeading___Toc402443273)

[8.3 STATISTICAL ANALYSIS PLAN 17](#__RefHeading___Toc402443274)

[9 ADVERSE EVENTS 17](#__RefHeading___Toc402443275)

[9.1 DEFINITION 17](#__RefHeading___Toc402443276)

[9.1.1 Adverse event (AE) 17](#__RefHeading___Toc402443277)

[9.1.2 Serious Adverse Event (SAE) 18](#__RefHeading___Toc402443278)

[9.1.3 Adverse Drug Reaction (ADR) 18](#__RefHeading___Toc402443279)

[9.1.4 Causality Assessment 18](#__RefHeading___Toc402443280)

[9.1.5 Severity of AEs 19](#__RefHeading___Toc402443281)

[9.2 MONITORING OF AEs 19](#__RefHeading___Toc402443282)

[9.2.1 Handling of AEs 20](#__RefHeading___Toc402443283)

[9.3 SUBMISSION OF NEW INFORMATION 20](#__RefHeading___Toc402443284)

[9.4 EXPECTED ADRs 20](#__RefHeading___Toc402443285)

[10 ETHICAL CONSIDERATIONS 21](#__RefHeading___Toc402443286)

[10.1.1 COMPLIANCE WITH ETHICAL PRINCIPLES 21](#__RefHeading___Toc402443287)

[10.2 INSTITUTIONAL REVIEW BOARD (IRB) 21](#__RefHeading___Toc402443288)

[10.3 INFORMED CONSENT 21](#__RefHeading___Toc402443289)

[10.3.1 When and How to Obtain Informed Consent 21](#__RefHeading___Toc402443290)

[10.3.2 Elements of Informed Consent 22](#__RefHeading___Toc402443291)

[10.3.3 Approval and Revisions of Informed Consent 23](#__RefHeading___Toc402443292)

[10.4 CONFIDENTIALITY 23](#__RefHeading___Toc402443293)

[10.5 SUBJECT STIPEND 23](#__RefHeading___Toc402443294)

[10.6 HEALTH DAMAGE COMPENSATION AND INSURANCE 24](#__RefHeading___Toc402443295)

[10.7 STUDY COMPLETION OR PREMATURE TERMINATION AND SUSPENSION 24](#__RefHeading___Toc402443296)

[10.7.1 Study Completion 24](#__RefHeading___Toc402443297)

[10.7.2 Criteria for Study Termination or Suspension 25](#__RefHeading___Toc402443298)

[10.7.3 Procedures for Premature Termination or Suspension 25](#__RefHeading___Toc402443299)

[11 PROTOCOL COMPLIANCE 25](#__RefHeading___Toc402443300)

[11.1 PROTOCOL COMPLIANCE 25](#__RefHeading___Toc402443301)

[11.2 PROTOCOL DEVIATION(S) AND CHANGE(S) 25](#__RefHeading___Toc402443302)

[11.3 PROTOCOL AMENDMENT(S) 26](#__RefHeading___Toc402443303)

[12 DATA COLLECTION AND STORAGE 26](#__RefHeading___Toc402443304)

[12.1 CASE REPORT FORM (CRF) 26](#__RefHeading___Toc402443305)

[12.2 STORAGE OF RECORDS 26](#__RefHeading___Toc402443306)

[13 SOURCE DOCUMENT VERIFICATION 27](#__RefHeading___Toc402443307)

[13.1 SOURCE DOCUMENT SPECIFICATIONS 27](#__RefHeading___Toc402443308)

[13.2 PROCEDURES FOR DIRECT ACCESS 27](#__RefHeading___Toc402443309)

[13.3 EVALUATION AND HANDLING OF VERIFICATION OF RESULTS 27](#__RefHeading___Toc402443310)

[14 QUALITY CONTROL AND QUALITY ASSURANCE 28](#__RefHeading___Toc402443311)

[14.1 QUALITY CONTROL 28](#__RefHeading___Toc402443312)

[14.2 QUALITY ASSURANCE 28](#__RefHeading___Toc402443313)

[15 PUBLICATION POLICY 28](#__RefHeading___Toc402443314)

[16 STUDY PERIOD 28](#__RefHeading___Toc402443315)

[17 STUDY ORGANIZATION 28](#__RefHeading___Toc402443316)

[17.1 SPONSOR 28](#__RefHeading___Toc402443317)

[17.1.1 Sponsor 28](#__RefHeading___Toc402443318)

[17.1.2 Sponsor`s Study Organization 29](#__RefHeading___Toc402443319)

[17.1.3 Medical Advisor 29](#__RefHeading___Toc402443320)

[17.2 TESTING FACILITY FOR TJ-54 CONSITUENTS IN PLASMA 29](#__RefHeading___Toc402443321)

[17.3 RESEARCH FACILITY AND PRINCIPAL INVESTIGATOR 29](#__RefHeading___Toc402443322)

[18 REFERENCES 29](#__RefHeading___Toc402443323)

# STUDY SYNOPSIS

## TITLE

Pharmacokinetic study of single oral administration of yokukansan (YKS: TJ-54) in healthy volunteers

## STUDY OBJECTIVE

To evaluate the pharmacokinetics and safety of Tsumura Yokukansan Extract Granules for prescription (hereinafter referred to as “TJ-54”)after single oral doses of 7.5g （1 sachet 2.5g x 3 sachets）, 5.0g （1 sachet 2.5g x 2 sachets）, and 2.5g （1 sachet 2.5g x 1 sachet） in healthy volunteers

## ENDPOINTS

Pharmacokinetics and safety assessment

## STUDY POPULATION

### Subjects

Healthy volunteers

### Target Sample Size to Complete Study

At least 15 subjects

### Inclusion Criteria

1) Age 20-44 years, inclusive (at the time of informed consent)

2) Subject body mass index (BMI) is between 18.5 and 25 kg/m^2^

3) Genders: Both

4) Willing and able to provide written, signed informed consent

### Exclusion Criteria

1) Subjects with a history of allergic reactions to drugs (including Kampo medicines) and food(s).

2) Female subjects who are pregnant, breastfeeding or planning to become pregnant.

3) Subjects who have participated in another clinical trial within the past 16 weeks.

4) Subjects who have had their blood drawn or donated > 400mL of blood within 12 weeks of study drug administration.

5) History of significant hepatic, cardiovascular or hematological disease.

6) Subjects with hepatic steatosis.

7) Subjects who can’t stop taking of alcohol and/or tobacco products within 3 days of study medication dosing.

8) Use of any medications (e.g., antipsychotic, antifungal, antihypertensive, Kampo medicine, etc.) within 7 days prior to study drug administration.

9) Use of supplements containing atractylodes lancea rhizome, poria sclerotium, cnidium rhizome, uncaria hook, Japanese angelica root, bupleurum root, and/or glycyrrhiza within 3 days of study medication dosing.

10) Use of any agent within 7 days prior to study drug administration.

11) Subjects with abnormal clinical laboratory values inappropriate for the study in the opinion of the principal investigator/subinvestigator.

12) Subjects who are deemed ineligible for other reasons by the principal investigator/subinvestigator.

13) Subjects of HCV antibody, HBs antigen, and/or HIV antibody positive.

## STUDY DESIGN

Randomized crossover study

## DOSAGE AND ADMINISTRATION

Single oral administration of TJ-54 of 7.5g (1 sachet 2.5g x 3 sachets), 5.0g (1 sachet 2.5g x 2 sachets), and 2.5g (1 sachet 2.5g x 1 sachet).

## SCHEDULE OF TIME AND EVENTS

Subjects will be randomly assigned to Groups A – C after enrollment, and three different dosages will be administered to the same subject at three separate times. The three dosage periods will be designated as phase I, phase II and phase III with a washout period of greater than 4 weeks between each treatment period, i.e., between phases I and II, and phases II and III (Table 1).

Table 1 Dosing Schedule

|  | Phase I | Washout period | Phase II | Washout period | Phase III |
| --- | --- | --- | --- | --- | --- |
| Gp A | 7.5g | > 4 weeks | 2.5g | > 4 weeks | 5.0g |
| Gp B | 2.5g | > 4 weeks | 5.0g | > 4 weeks | 7.5g |
| Gp C | 5.0g | > 4 weeks | 7.5g | > 4 weeks | 2.5g |

## OTHER FACTORS FOR CONTROL

1) Supplements containingg atractylodes lancea rhizome, poria sclerotium, cnidium rhizome, uncaria hook, Japanese angelica root, bupleurum root, and/or glycyrrhiza are strictly prohibited from 3 days prior to dispensing the study medication until completion of each treatment phase. In addition, foods and drinks containing caffeine, alcohol, carbonated water, glycyrrhiza (that there is a notation in ingredients), garlic and citrus fruits (grapefruit, orange, tangerine, etc.) are prohibited from 3 days prior to dispensing the study medication until completion of each treatment phase. Meals must be consumed before 20:00 on the eve of study drug administration, and subjects must have fasted from 20:00 until the postdose, 4-hour sampling is complete. However, water may be consumed at any time.

2) Smoking is prohibited within 7 days prior to dosing study medication until the completion of each treatment phase of the study.

3) Use of other medications is prohibited within 7 days prior to dosing study medication until the completion of each treatment phase of the study.

## STUDY PERIOD

May 2012 – December 2012

## INSTITUTION

Kochi Medical School Hospital

Kohasu Oko-cho, Nankoku-shi, Kochi 783-8505, Japan

TEL: +81-88-866-5811

## SPONSOR

Tsumura & Co. 2-17-11 Akasaka Minato-ku, Tokyo 107-8521, Japan

Contact information: Kampo Research Planning Department

TEL: +813-6361-7185 FAX: +813-5574-6664

# BACKGROUND

Yokukansan, a classical herbal prescription, is composed of seven crude drugs, namely, atractylodes lancea rhizome, poria sclerotium, cnidium rhizome, uncaria hook, Japanese angelica root, bupleurum root, and glycyrrhiza. TJ-54 is yokukansan manufactured as convenient granules via Tsumura`s proprietary dry granulation method and officially approved as Kampo extract formulation for prescription under the provisions stipulated in “Report No. 120 of the Central Pharmaceutical Affairs Council, Ministry of Health and Welfare dated May 31, 1985”. TJ-54 has broad clinical applications for the treatment of insomnia^1)^, peevishness in children, and behavioral and psychological symptoms of dementia^2)-5)^. Reported pharmacological effects of TJ-54 ameliorated aggressiveness^6)-9)^. Recently, several basic studies have clarified that the serotonergic nervous^9)-11)^ and glutamatergic^12)-15)^ systems are associated with the mechanisms of TJ-54.

While reports on the pharmacological effects of TJ-54 abound, pharmacokinetic studies of TJ-54 are difficult to execute due to complexity of multiple constituents. Although pharmacokinetic parameters of single isolated constituents are occasionally seen in literatures, pharmacokinetic investigation of TJ-54 as a compound prescription in animal and human studies is insubstantial^16)^.

The “Preliminary absorption study of yokukansan (YKS:TJ-54)in healthy volunteers TJ-54 (TJ-54-4-2)” was conducted from April 2011 to identify constituents that were absorbed after a single oral dose of 7.5g of TJ-54 in healthy adults^17)^. From the results of the previous preliminary absorption study, 3 compounds of pharmacological actions have been suggested in TJ-54 (geissoschizine methyl ether^11), 18)-20)^, hirsuteine^20)^, and 18β-glycyrrhetinic acid^13)^) were confirmed in plasma.

The reports of licorice is one of the configuration crude drugs TJ-54, the long-term high intake are likely to develop pseudoaldosteronism, there is suggested 18β-glycyrrhetinic acid and glycyrrhetinic acid 3-O-glucuronide are causal compounds^21)^. Therefore, this study also confirms the plasma concentration of glycyrrhetinic acid 3-O-glucuronide. This finding is believed to provide important data for the side effect studies.

The objective of this study is to evaluate the pharmacokinetics and safety of TJ-54 by measuring the plasma concentration of four TJ-54 derivative compounds.

The present study will be conducted in accordance with the trial protocol and standards specified under the MHLW ordinance regarding Good Clinical Practice (GCP), MHLW ordinance regarding Good Post-Marketing Surveillance/Study Practice (GPSP), and Article 14, Paragraph 3, and Article 80, Paragraph 2 of the Pharmaceutical Affairs Law.

# STUDY OBJECTIVE

## Study Objective

To evaluate the pharmacokinetics and safety of Tsumura Yokukansan Extract Granules for prescription (hereinafter referred to as “TJ-54”)after single oral doses of 7.5g (1 sachet 2.5g x 3 sachets), 5.0g (1 sachet 2.5g x 2 sachets), and 2.5g (1 sachet 2.5g x 1 sachet) in healthy volunteers.

## TYPE OF STUDY

Post-marketing clinical trial

# STUDY POPULATION

## Subjects

Healthy adult volunteers

## Target Sample Size

At least 15 completed subjects

[Rationale for Sample Size Determination]

15 subjects have been determined according to the conventional sample size required to evaluate pharmacokinetics. For a definition of the term “completed”, refer to 5.3.1.

## Inclusion Criteria

1) Japanese

2) Age 20-44 years, inclusive (at the time of informed consent)

3) Subject body mass index (BMI) is between 18.5 and 25 kg/m^2^

4) Genders: Both

5) Subject is willing and able to provide written informed consent

[Rationale for Inclusion Criteria]

This study is directed to Japanese. The purpose of setting the lower age limit is to select adults with the ability to make an informed decision about study participation, while the upper age limit serves to dampen physiological effects of aging from older subjects. Body mass index (BMI) within normal range as specified by the Japan Society for the Study of Obesity (JASSO) is included to minimize the effects of obesity on the distribution of the study medication.

## Exclusion Criteria

1) Subjects with a history of allergic reactions to drugs (including Kampo medicines) and food(s).

2) Female subjects who are pregnant, breastfeeding or planning to become pregnant.

3) Subjects who have participated in another clinical trial within the past 16 weeks.

4) Subjects who have had their blood drawn or donated > 400mL of blood within 12 weeks of study drug administration.

5) History of significant hepatic, cardiovascular or hematological disease.

6) Subjects with hepatic steatosis.

7) Subjects who can’t stop taking of alcohol and/or tobacco products within 3 days of study medication dosing.

8) Use of any medications (e.g., antipsychotic, antifungal, antihypertensive, Kampo medicine, etc.) within 7 days prior to study drug administration.

9) Use of supplements containing atractylodes lancea rhizome, poria sclerotium, cnidium rhizome, uncaria hook, Japanese angelica root, bupleurum root, and/or glycyrrhiza within 3 days of study medication dosing.

10) Use of any agent within 7 days prior to study drug administration.

11) Subjects with abnormal clinical laboratory values inappropriate for the study in the opinion of the principal investigator/subinvestigator.

12) Subjects who are deemed ineligible for other reasons by the principal investigator/subinvestigator.

13) Subjects of HCV antibody, HBs antigen, and/or HIV antibody positive.

[Rationale for Exclusion Criteria]

1)-5) ensure the safety of subjects, 6)-12) eliminate subjects who may interfere with the objectives of the study, and 13) ensure the safety of analysts.

# STUDY METHOD

## STUDY DESIGN

This is a randomized crossover study. Study medication will be administered to the same subjects after a washout period of greater than 4 weeks.

## DOSAGE AND ADMINISTRATION

Subjects will be randomly assigned to Groups A – C after enrollment, and three different dosages will be administered to the same subject at three separate times. The three dosage periods will be designated as phase I, phase II and phase III with a washout period of greater than 4 weeks between each treatment period, i.e., between phases I and II, and phases II and III (Table 1).

Table 1 Dosing Schedule

|  | Phase I | Washout period | Phase II | Washout period | Phase III |
| --- | --- | --- | --- | --- | --- |
| Gp A | 7.5g | > 4 weeks | 2.5g | > 4 weeks | 5.0g |
| Gp B | 2.5g | > 4 weeks | 5.0g | > 4 weeks | 7.5g |
| Gp C | 5.0g | > 4 weeks | 7.5g | > 4 weeks | 2.5g |

## STUDY COMPLETION; SUBJECT DISCONTINUATION, WITHDRAWAL OR REPLACEMENT

### Study Completion

Each treatment period will be considered complete after the 48-hour blood sampling and testing at the end of the period.

The study will conclude after the completion of the 3 treatment phases.

### Subject Discontinuation or Withdrawal

The investigator may terminate or withdraw a subject from the study if the subject meets any of the following criteria:

1) Requests withdrawal or declines to participate;

2) Experiences an adverse events (AE) that undermines the continuation of the study in the opinion of the principal investigator;

3) Unable to take the study medication;

4) Judged as ineligible to continue the study by the principal investigator.

### Discontinuation Procedure

The principal investigator/subinvestigator must document subjects who discontinue the study and the reason(s) for it, measures taken and follow-up in the Case Record Form (CRF). If discontinuation is for safety reasons, appropriate measures must be taken and subjects should be followed to resolution and receive follow-up evaluations until their safety is assured, except in cases where consent cannot be obtained from the subjects.

### MANAGEMENT OF SUBJECTS

All subjects are under close supervision of the principal investigator/subinvestigator during their participation in the clinical trial.

The principal investigator/subinvestigator will instruct all subjects to comply with the following directions.

1) Supplements containingg atractylodes lancea rhizome, poria sclerotium, cnidium rhizome, uncaria hook, Japanese angelica root, bupleurum root, and/or glycyrrhiza are strictly prohibited from 3 days prior to dispensing the study medication until completion of each treatment phase. In addition, foods and drinks containing caffeine, alcohol, carbonated water, glycyrrhiza (that there is a notation in ingredients), garlic and citrus fruits (grapefruit, orange, tangerine, etc.) are prohibited from 3 days prior to dispensing the study medication until completion of each treatment phase. Meals must be consumed before 20:00 on the eve of study drug administration, and subjects must have fasted from 20:00 until the postdose, 4-hour sampling is complete. However, water may be consumed at any time.

2) Smoking is prohibited within 7 days prior to dosing study medication until the completion of each treatment phase of the study.

3) Use of other medications is prohibited within 7 days prior to dosing study medication until the completion of each treatment phase of the study.

# CLINICAL TRIAL MATERIAL (CTM)

## PROPRIETARY NAME AND MANUFACTURER

Study medication: Tsumura Yokukansan Extract Granule (for prescription)

Code number: TJ-54

Manufacturer: Tsumura & Co.

## CONSTITUENTS, DOSAGE FORM, STORAGE

Ingredients/Content: 7.5g of TJ-54 contains 3.25g of dried extract of the following ratio of crude drugs.

JP Atractylodes Lancea Rhizome ...... 4.0g

JP Poria Sclerotium ............................ 4.0g

JP Cnidium Rhizome .......................... 3.0g

JP Uncaria Hook …............................ 3.0g

JP Japanese Angelica Root ................. 3.0g

JP Bupleurum Root ............................ 2.0g

JP Glycyrrhiza .................................... 1.5g

(JP: The Japanese Pharmacopoeia)

Excipients: JP Magnesium stearate, JP Lactose Hydrate

Dosage form: Extract granule

Packaging: One heat-sealed sachet contains 2.5g of extract granule

Storage: Store in cool, dry place. Avoid contact with moisture and direct sunlight.

## DOSAGE AND ADMINISTRATION

Single oral administration of TJ-54 of 7.5g (1 sachet 2.5g x 3 sachets), 5.0g(1 sachet 2.5g x 2 sachets), and 2.5g(1 sachet 2.5g x 1 sachet).

## SUPPLY AND DELIVERY

The clinical trial material (CTM) will be supplied and delivered by the sponsor to the institution for free after the study contract is signed between the two parties.

## STORAGE AND HANDLING

The study drug administrator will store and handle the CTM in appropriate manner.

## RETRIEVAL

The sponsor will retrieve all unused CTM after inspection by the sponsor.

# MEASUREMENTS AND EVALUATIONS

## INSPECTION AND OBSERVATION ITEMS

### Screening Items

The principal investigator/subinvestigator will conduct the following medical interview, clinical observations and laboratory tests within 7 days prior to the start of the trial, and select prospective subjects based on their results. Tolerance screening date is ±1 day.

Demographics: date of birth (DOB), gender, race, present illness, current medications, drug and food allergies, past medical history, alcohol intake, smoking history

Clinical findings: subjective symptoms, objective findings, edema

Physical examination: height, weight, BMI, body temperature (axillary), blood pressure (seated), pulse (seated)

Laboratory tests:

1) Hematology

Erythrocytes, leukocytes, platelets, hemoglobin, hematocrit

2) Blood chemistry

Total protein, blood urea nitrogen (BUN), creatinine, uric acid, aspartate transaminase (AST), alanine aminotransferase (ALT), total bilirubin (T-Bil), alakaline phosphatase (ALP), gamma-glutamyl transpeptidase (γ-GTP), albumin (Alb), prothrombin time (PT), total cholesterol (TC), C-reactive protein (CRP), Hb_A1C_, kalium (K)

3) Urinalysis

Urinary glucose

Urinary Pregnancy test (females only)

Viral tests: HCV antibody, HBs antigen, HIV antibody

### Predose Tests

The principal investigator/subinvestigator will perform the following clinical observations and laboratory tests within one day prior to dispensing the study medication, and select prospective subjects based on the comprehensive analysis of test results.

Clinical findings: subjective symptoms, objective findings, edema

Physical examination: body temperature (axillary), blood pressure (seated), pulse (seated)

Laboratory tests:

1) Hematology

Erythrocytes, leukocytes, platelets, hemoglobin, hematocrit

2) Blood chemistry

Total protein, blood urea nitrogen (BUN), creatinine, uric acid, aspartate transaminase (AST), alanine aminotransferase (ALT), total bilirubin (T-Bil), alakaline phosphatase (ALP), gamma-glutamyl transpeptidase (γ-GTP), albumin (Alb), prothrombin time (PT), total cholesterol (TC), C-reactive protein (CRP), Hb_A1C_, kalium (K)

Urinary Pregnancy test (females only): Phase I only, inspection at the time of screening.

### Test at the Administration

Clinical findings: subjective symptoms, objective findings

### Tests at the End of Study

At the end of each treatment phase, the principal investigator/subinvestigator will perform the following clinical observations and laboratory tests.

Clinical findings: subjective symptoms, objective findings, edema

Physical examination: body temperature (axillary), blood pressure (seated), pulse (seated)

Laboratory tests:

1) Hematology

Erythrocytes, leukocytes, platelets, hemoglobin, hematocrit

2) Blood chemistry

Total protein, blood urea nitrogen (BUN), uric acid, aspartate transaminase (AST), alanine aminotransferase (ALT), total bilirubin (T-Bil), alkaline phosphatase (ALP), gamma-glutamyl transpeptidase (GTP), albumin (Alb), prothrombin time (PT), total cholesterol, C-reactive protein (CRP), kalium (K)

### Search of TJ-54 Constituents in the Plasma

The total amount of sampling will be 368mL (Screening: 8mL x 1, 120mL x 3 phases {(Clinical laboratory: 8mL x 2 times, Pharmacokinetics: 8mL x 13 times)}x3).

Blood collection:

**i) Sampling times**

Predose sample and postdose samples at 15 min, 30 min, 1, 2, 3, 4, 8, 10, 12, 14, 24, and 48-hour will be collected(13 total samples).

**ii) Method and handling of sampling**

A total of 8mL of venous blood drawn from the subject`s antecubital vein during the sampling time windows will be evacuated into a tube (8mL for pharmacokinetic study: heparin sodium as anticoagulant), and centrifuged at 1700 × g for 10 minutes at 4 °C to obtain the plasma. The plasma obtained from the blood collection tube containing 8ml pharmacokinetic sample will be dispensed into tubes containing 1 mL each and cryopreserved below -20 °C until analysis.

**iii) Acceptable range and handling of sampling time error**

Sampling times will be recorded at the start of collection. The acceptable sampling time windows for postdose collection at 15 min, 30 min, and 1-hour will be set within 10% of the sampling times (15 min + 1.5 min, 30 min + 3 min, 1-hour + 6 min). The acceptable sampling windows for the 2, 3, and 4-hour collection will be uniformly set within + 10 min of collection (2-hour + 10 min, 3-hour + 10 min, 4-hour + 10 min). The acceptable sampling windows for 8, 10, 12, 14, 24 and 48-hour collection will be uniformly set within + 30 min of collection (8-hour + 30 min, 10-hour + 30 min, 12-hour + 30 min, 14-hour + 30 min, 24-hour + 30 min, 48-hour + 30 min).

Deviations from sampling time windows will be indicated in the CRF. Samples obtained for analysis may be submitted as well. The handling of analyzed data should follow the separately prepared pharmacokinetic analysis protocol.

### Safety Parameters

Adverse events

## SCHEDULE OF TIME AND EVENTS

This randomized crossover study will be executed as three treatment phases (Table 2).

Table 2 Study Schedule 1 (from start to completion)

|  | Day-7 | Phase I | | >4 we. | Phase II | | >4 we. | Phase III | |
| --- | --- | --- | --- | --- | --- | --- | --- | --- | --- |
|  |  | Day-1 | Day-0 to Day+1 | Washout Period | Day-1 | Day-0 to Day+1 | Washout Period | Day-1 | Day-0 to Day+1 |
|  | Screening | Predose | Dosing and  blood sampling  (until 24h) |  | Predose | Dosing and  blood sampling  (until 24h) |  | Predose | Dosing and  blood sampling  (until 24h) |
| Informed consent | 〇 |  |  |  |  |  |  |  |  |
| Enrollment |  | 〇 |  |  |  |  |  |  |  |
| Allocation |  |  | 〇 |  |  |  |  |  |  |
| Demographics | 〇 |  |  |  |  |  |  |  |  |
| Ht, Wt, BMI | 〇 |  |  |  |  |  |  |  |  |
| BP, Pulse, BT | 〇 | 〇 | 〇 |  | 〇 | 〇 |  | 〇 | 〇 |
| Clinical exam | 〇 | 〇 | 〇 |  | 〇 | 〇 |  | 〇 | 〇 |
| Urinary pregnancy test | 〇 |  |  |  | 〇 |  |  | 〇 |  |
| Lab tests | 〇 | 〇 | 〇 |  | 〇 | 〇 |  | 〇 | 〇 |
| TJ-54 gps  Gp A  Gp B  Gp C |  |  | 〇  7.5g  2.5g  5g |  |  | 〇  2.5g  5g  7.5g |  |  | 〇  5g  7.5g  2.5g |
| PK study |  |  | 〇 |  |  | 〇 |  |  | 〇 |
| Safety  Assessment |  |  |  |  |  |  |  |  |  |

Each laboratory test and clinical observation will be performed according the study schedule in Table 3 (sample description of schedule). As a rule, subjects will be admitted one day at the start of study medication, released upon completion of 24-hour blood draw, and expected to return to the hospital for 48-hour sampling.

Table 3 Study Schedule 2 (Phase I) ^Note 1^

|  | Visiting | | Admission | | | | | | | | | | | | | | Visiting |
| --- | --- | --- | --- | --- | --- | --- | --- | --- | --- | --- | --- | --- | --- | --- | --- | --- | --- |
|  | Day  -7 | Day  -1 | Day-0 | | | | | | | | | | | | Day  +1 | Day  +2 | |
|  | Screening | Day before dosing | Immediately before dosing | 0  hr | 15  min | 30  min | 1 hr | 2 hr | 3 hr | 4 hr | 8 hr | 10  hr | 12  hr | 14  hr | 24  hr | 48  hr | |
|  |  |  | 7:30 | 8:00 | 8:15 | 8:30 | 9:00 | 10:00 | 11:00 | 12:00 | 16:00 | 18:00 | 20:00 | 22:00 | 8:00 | 8:00 | |
| Informed consent | ○ |  |  |  |  |  |  |  |  |  |  |  |  |  |  |  | |
| Subject roster |  | ○ |  |  |  |  |  |  |  |  |  |  |  |  |  |  | |
| Allocation |  |  | ○ |  |  |  |  |  |  |  |  |  |  |  |  |  | |
| Demographics | ○ |  |  |  |  |  |  |  |  |  |  |  |  |  |  |  | |
| Height, Weight  BMI | ○ |  |  |  |  |  |  |  |  |  |  |  |  |  |  |  | |
| BP, Pulse  BT | ○ | ○ |  |  |  |  |  |  |  |  |  |  |  |  |  | ○ | |
| Clinical exam | ○ | ○ | ○ |  |  |  |  |  |  |  |  |  |  |  |  | ○ | |
| Urinary pregnancy test^Note2^ | ○ | |  |  |  |  |  |  |  |  |  |  |  |  |  |  | |
| Lab tests ^Note3^  sample volume | ○  8mL | ○  8mL |  |  |  |  |  |  |  |  |  |  |  |  |  | ○  8mL | |
| TJ-54 dosing |  |  |  | ○ |  |  |  |  |  |  |  |  |  |  |  |  | |
| Meal^Note4^ |  | ○ |  |  |  |  |  |  |  | ○ |  | ○ |  |  | ○ | ○ | |
| PK ^Note3^  sample volume |  |  | ○  8mL |  | ○  8mL | ○  8mL | ○  8mL | ○  8mL | ○  8mL | ○  8mL | ○  8mL | ○  8mL | ○  8mL | ○  8mL | ○  8mL | ○  8mL | |
| Safety assessment |  |  |  |  |  |  |  |  |  |  |  |  |  |  |  |  | |

Note 1: The above schedule is for Phase I. Screening tests conducted 7 days prior to dosing of study medication will not be performed in Phases II and III, i.e., predose tests one day before dosing and other tests scheduled thereafter will be performed.

Note 2: Conduct in females only. Urinary pregnancy test is performed at the time of screening tests in Phase I, the Phases II and III are carried out the day before drug dosing of study medication.

Note 3: The total blood draw will be 368mL [screening: 8mL x 1, 120mL x 3 phases {(8mL x 2 for laboratory tests, 8ml x 13 for pharmacokinetic) x 3}]. In addition, the 4, 12, 24, and 48-hour sampling will be collected before meal.

Note 4: Supplements containingg atractylodes lancea rhizome, poria sclerotium, cnidium rhizome, uncaria hook, Japanese angelica root, bupleurum root, and/or glycyrrhiza are strictly prohibited from 3 days prior to dispensing the study medication until completion of each treatment phase. In addition, foods and drinks containing caffeine, alcohol, carbonated water, glycyrrhiza (that there is a notation in ingredients), garlic and citrus fruits (grapefruit, orange, tangerine, etc.) are prohibited from 3 days prior to dispensing the study medication until completion of each treatment phase. Meals must be consumed before 20:00 on the eve of study drug administration, and subjects must have fasted from 20:00 until the postdose, 4-hour sampling is complete. However, water may be consumed at any time.

# CRITERIA AND ANALYSIS FOR EVALUATION

## TARGET POPULATION

### Target Population for Pharmacokinetic Analysis

Subjects who have completed sampling for pharmacokinetic analysis after dosing of study medication.

### Target Population for Safety Analysis

Subjects who have taken the study medication.

## ENDPOINTS

### Pharmacokinetics

Pharmacokinetics will be evaluated using the collected blood samples to calculate pharmacokinetic parameters: maximum drug concentration in plasma (C_max_) obtained from plasma concentration; maximum drug concentration time in plasma (t_max_); and area under the blood concentration-time curve (AUC) of the 4 compounds (geissoschizine methyl ether, hirsuteine, 18β-glycyrrhetinic acid, and glycyrrhetinic acid 3-O-glucuronide) of which validated analytical method have been established.

The details of plasma concentration analysis of the 4 compounds will depend on the separately prepared study protocol. Sekisui Medical will perform the analyses; however, additional analyses may be outsourced as needed.

The details of pharmacokinetic analyses such as linear analysis and pharmacokinetic parameters will be documented in a separate pharmacokinetic analysis plan. Tsumura Laboratories will perform statistical analyses of pharmacokinetic data by following this plan. Data from blood collected outside the sampling windows will be handled as described in the pharmacokinetic analysis plan.

Various pharmacokinetic data including measured plasma concentration may be used for a separate analysis at a later time.

The content of the 4 compounds in TJ-54 drug lots for pharmacokinetic analysis will be obtained from plasma concentration analysis of the TJ-54 lot used by Sekisui Medical.

### Safety assessment

Safety will be determined from the overall assessment of adverse events, clinical findings, physical examination, and laboratory results. As a rule, the average of screening and predose test results will become baseline values (test results obtained exclusively from screening will be used as they are) and changes observed after dosing will be taken into account.

### Handling of Subjects

Based on Tsumura-GCP/SOP/11, the sponsor will establish the target population for pharmacokinetic-related analysis and safety-related analysis as described above.

## STATISTICAL ANALYSIS PLAN

Identifying and classifying the endpoints for analysis, as well as the timeframe and methods for conducting analyses should follow the “Statistical Analysis Plan (Form 2)”. Further, statistical analysis will be performed after the guideline for statistical analysis is generated.

# ADVERSE EVENTS

## DEFINITION

### Adverse event (AE)

An adverse event (AE) is any unfavorable or unintended medical occurrence (including abnormal laboratory values), symptom or disease in a subject following exposure to study agent, regardless of causal attribution to the study medication. An event that develops during the study or any event already present that worsens in either intensity or frequency is included as well.

### Serious Adverse Event (SAE)

A serious adverse event is defined as an AE that results in one of the following outcomes:

(1) Death

(2) Immediately life-threatening

(3) Requires inpatient hospitalization or prolongation of existing hospitalization

(4) Results in persistent or significant disability/incapacity

(5) Is a congenital anomaly or birth defect

(6) Other medically significant event

### Adverse Drug Reaction (ADR)

An adverse drug reaction is an event for which a causal relationship to the study medication cannot be ruled out (refers to 1) – 3) of 9.1.4 Causality Assessment).

### Causality Assessment

The following criteria will be used to characterize causality into four classifications:

[Causality Classification]

1) Definitely Related

There is evidence to suggest a plausible temporal relationship (including follow-up after termination of treatment) between the study medication and the AE, as well as solid facts and sufficient evidence to support the relationship.

2) Probably Related

There is evidence to suggest a reasonable temporal relationship (including follow-up after termination of treatment) between and the study medication and the AE, and is unlikely to be attributed to other factors such as underlying disease, concurrent illness, and concurrent medication.

3) Possibly Related

There is evidence to suggest a reasonable temporal relationship (including follow-up after termination of treatment) between the study medication and the AE. However, factors such as underlying disease, concurrent illness, concurrent medication/intervention may also be implicated.

4) Not Related

The temporal relationship between the study medication and the AE is improbable because other factors such as underlying disease, concurrent illness, concurrent medication/intervention provide plausible explanations.

### Severity of AEs

1) Mild:

The event is generally temporary, does not impair the person`s ability to conduct normal life functions, and does not require treatment (normal activities are possible).

2) Moderate

The event interferes with the person`s ability to conduct normal life functions, causes considerable discomfort, and requires treatment (normal activities are accompanied by discomfort).

3) Severe

The event severely impairs the person`s ability to conduct normal life functions and requires treatment (normal activities are onerous).

## MONITORING OF AEs

Any AE must be investigated through inquiry and observation, and if confirmed, the event must be followed and details documented in the CRF.

i) type of adverse event

ii) date of onset

iii) seriousness (1. non-serious, 2. serious)

iv) rationale for seriousness and other medically significant events

v) severity (1. mild, 2. moderate, 3. severe)

vi) treatment (description of intervention if implemented)

vii) outcome (1. recovery (resolution), 2. remission, 3. irresolution (unchanged), 4. complication, 5. unknown)

viii) date of verified outcome

ix) causality (1. definitely related, 2. probably related, 3. possibly related, 4. not related)

The principal investigator/subinvestigator must document clinically significant, abnormal changes in laboratory values and their explanations in the CRF.

The causality assessment between the AE and the study medication must be documented in the CRF.

### Handling of AEs

Regardless of causality, the principal investigator must promptly (within 24 hours) report any AE considered serious based on the criteria in section 9.1.2 to the affiliated institution head/IRB and sponsor in person, by telephone or fax, take appropriate actions, and submit a written “Serious Adverse Event Report Form (Form 3)” within few days of the event. In addition, the principal investigator/subinvestigator must provide additional information to the sponsor, institution head and IRB upon request.

The institution head must seek IRB`s assessment of the institution`s competence to continue the study.

The sponsor must immediately report expected ADRs and unexpected serious or non-serious ADRs from precautions in suspected subjects to appropriate regulatory agencies in accordance with Tsumura-GCP/SOP and Tsumura-GSPS/SOP, as well as Article 77, Paragraph 4, Item 2 and Article 253 of the Pharmaceutical Affairs Act.

## SUBMISSION OF NEW INFORMATION

Upon receipt of new information regarding product document revisions and safety of study medication, the sponsor must promptly notify the institution head/IRB and the principal investigator in writing, and take appropriate measures.

## EXPECTED ADRs

Refer to the product document “Tsumura Yokukansan Extract Granule (Prescription) Package Insert”.

# ETHICAL CONSIDERATIONS

### COMPLIANCE WITH ETHICAL PRINCIPLES

This study will be conducted in accordance with the spirit of the Declaration of Helsinki, study protocol, standards specified under the Pharmaceutical Affairs Act Article 80, Paragraph 2, Good Clinical Practice (GCP) effective as of April 1, 1997 (Ministry of Health and Welfare Ordinance No. 28), and related ministerial ordinances and notifications.

In addition, the study protocol will be periodically revised if necessary.

## INSTITUTIONAL REVIEW BOARD (IRB)

Prior to the beginning of the study, the IRB of the institution will examine the study protocol, CRF format, elements of the written informed consent form, and adequacy of study duration.

## INFORMED CONSENT

### When and How to Obtain Informed Consent

The principal investigator/subinvestigator will clarify the elements of 10.3.2 based on the explanatory statement to study participants one week prior to study drug administration, and obtain their freely given written agreement after verifying their understanding. The written informed consent form will be sealed or signed and personally dated by the subject and the principal investigator/subinvestigator who will conduct the consent discussion. In addition to documenting this information in the CRF, consent forms will be submitted to the department assigned by the institution or attached to medical records, and copies of the consent form including the explanatory statement will be provided to all subjects.

The principal investigator/subinvestigator will inform the subjects in a timely manner if information becomes available that may be relevant to the subject`s willingness to continue participation in the study. Upon ascertaining the subject`s continued interest, a written verification thereof will be dated and recorded.

### Elements of Informed Consent

The explanatory statement (of the informed consent form) will be used to explain the following content of the study to the subjects.

(1) That the study involves research.

(2) The purpose of the study (including explanation of dietary restrictions).

(3) The name, title and contact information of the principal investigator.

(4) The study procedures to be followed (experimental aspects, selection criteria).

(5) The reasonably expected benefits and foreseeable risks or inconveniences to the subject (when there is no intended clinical benefit to the subject, the subject should be made aware of this).

(6) The expected duration of the subject`s participation in the trial.

(7) That the subject`s participation in the trial is voluntary and that the subject may refuse to participate or withdraw from the study, at any time, without penalty or loss of benefits to which the subject is otherwise entitled.

(8) That the monitor(s), the auditor(s), the IRB/IEC, and the regulatory authority(ies) will be granted direct access to the subject`s original medical records for verification of clinical study procedures and/or data, without violating the confidentiality of the subject, and that by sealing or signing the written informed consent form, the subject is authorizing such access.

(9) That records identifying the subject will remain confidential if the results of the study are published.

(10) The institution`s customer service number to contact to obtain further information regarding the study, rights of subjects and in the event of study-related injury.

(11) The compensation and treatment available to subjects in the event of study-related injury.

(12) The approximate number of subjects involved in the study.

(13) That the subject will be informed in a timely manner if information becomes available that may be relevant to the subject`s willingness to continue participation in the study.

(14) The foreseeable circumstances and/or reasons under which the subject`s participation in the study may be terminated.

(15) The anticipated expenses and reasons for them, if any, to the subject for participating in the study.

(16) The anticipated prorated payment, if any, to the subject for participating in the study (agreement on amount, etc.).

(17) The subject`s responsibilities.

### Approval and Revisions of Informed Consent

The principal investigator will prepare the written informed consent form and obtain approval from the IRB before the study is initiated.

The principal investigator must promptly notify the institution head or IRB in writing if new information on safety of study medication become available and implement appropriate measures. If the institution head or IRB deems necessary to revise the consent form, revisions must be promptly made.

The principal investigator/subinvestigator will reexplain the revised consent form to the subjects and obtain their signed voluntary consent relevant to their continued participation in the study.

The principal investigator/subinvestigator will document the date of reconsent in the CRF, and provide copies of the newly sealed or signed and dated consent forms with the explanatory statement to the subjects.

## CONFIDENTIALITY

The subject identification codes will be used to codify subjects in the CRF and protect their anonymity from third parties. Specifically, subject codes assigned during enrollment will be used in lieu of the subjects` names and initials for identification and reference. Presentation and publication of study results must protect the subjects` identities at all times.

## SUBJECT STIPEND

Based on relevant institutional regulations or deliberation between the institution and sponsor (Tsumura & Co.), subjects will be compensated for participation, including transportation fees, to resolve any inconvenience incurred. Method of payment will be decided between the institution and Tsumura & Co.

## HEALTH DAMAGE COMPENSATION AND INSURANCE

Medical treatment and other necessary interventions will be provided to subjects who incur study-related injury. The sponsor will take precautions such as enrolling in insurance to indemnify against claims arising from the trial. If health damage occurs and subjects are entitled to compensation as determined by the principal investigator and the sponsor, and if the institution and subjects request for compensation, the principal investigator and chief investigator, upon agreement, will immediately provide medical expenses, medical care and compensation in accordance with the Tsumura-GCP/SOP. However, medical expenses will only cover reimbursement for out-of-pocket fees and exclude insurance benefits, and medical care and compensation will be provided according to the workers` compensation if necessary.

## STUDY COMPLETION OR PREMATURE TERMINATION AND SUSPENSION

### Study Completion

The principal investigator will prepare and submit the final report containing the elements outlined below to the institution head at the end of the study in a timely manner. The institution head will subsequently send a notification of study completion to the sponsor.

(1) Submission date of final report

(2) Name and address of sponsor

(3) Title of study

(4) Names of principal investigator and subinvestigators

(5) Study duration

(6) Number of subjects

(7) Summary of study results

(8) Status of GCP compliance

### Criteria for Study Termination or Suspension

The entire study is subject to premature termination or suspension if any of the following events occur during the study.

(1) Serious adverse event(s) that undermines the continuation of the study.

(2) Frequent adverse event(s) that undermines the continuation of the study.

(3) Latest basic study results (including findings from abroad) other than the results from the present study suggest serious implications for the subjects.

(4) The sponsor decides to terminate or suspend the study for other reasons

### Procedures for Premature Termination or Suspension

If the study is forced to prematurely terminate or suspend due to any of the reasons stated above, the sponsor must promptly notify the reasons for termination or suspension in writing to the principal investigator and the institution head/IRB.

In addition, the principal investigator must promptly inform the subjects, implement necessary measures, document subjects` study-related data in the CRF and submit them to the sponsor.

# PROTOCOL COMPLIANCE

## PROTOCOL COMPLIANCE

The study will be executed in accordance with the study protocol approved by the principal investigator and subinvestigator.

## PROTOCOL DEVIATION(S) AND CHANGE(S)

In principle, deviations or changes to the study protocol are not permitted except when medically necessary or authorized by the institution head in accordance with the IRB decision.

The principal investigator/subinvestigator will document all deviations from the protocol, provide a detailed written explanation to the sponsor, and retain a copy of the original.

The principal investigator/subinvestigator may deviate or revise the protocol without prior written consent from the sponsor and approval from the IRB to eliminate immediate hazards to the subjects or for compelling medical reasons.

The principal investigator will promptly provide notification to the sponsor, institution head and IRB of any changes that may significantly impact the study or increase the risk for subjects.

## PROTOCOL AMENDMENT(S)

If protocol amendments are forced to implement after the study commences, the sponsor will notify the details of changes to the institution head and the principal investigator. Depending on the degree of changes, the institution head will report or request review from the IRB, and provide instructions based on the review to the principal investigator. As evidence of agreement between the sponsor and the principal investigator concerning the implemented amendments, both parties will seal, sign and date the revised protocol or a surrogate document.

# DATA COLLECTION AND STORAGE

## CASE REPORT FORM (CRF)

Upon completion of all clinical and laboratory evaluations, the primary principal investigator/subinvestigator of the study will promptly generate the CRF, review the content, and seal or sign in the physician`s signature section on the cover the CRF.

The principal investigator will invariably review the content and check for errors in the CRF prepared by the subinvestigator, and if acceptable, will seal or sign in the principal investigator section before submitting the CRF to the sponsor.

The seal in the CRF should be consistent with the one on the “List of Signatures and Seals (Form 4)” prepared beforehand.

## STORAGE OF RECORDS

The personnel responsible for the storage of records, as designated by the institution head, must retain the following essential documents for 3 years at the institution after formal discontinuation or completion of the study: informed consent documents, original records used for preparing CRF (medical records, laboratory data, etc.), IRB`s deliberation documents and records, documents related to study proposal and contract, and the table of study medication administration. If the records require transfer for any reasons, the sponsor must be notified of the new address.

# SOURCE DOCUMENT VERIFICATION

The principal investigator and the institution (study site) will permit direct access to all study-related documents such as source data for IRB, trial-related monitor and sponsor`s auditor, and during inspection by IRB and regulatory agencies.

## SOURCE DOCUMENT SPECIFICATIONS

Prior to the commencement of the study, the principal investigator and sponsor will prepare the “List of Source Documents (Form 5)” to jointly specify source documents that contain data for CRF, storage area and personnel responsible for storage.

However, all of the comments, abnormal changes in laboratory values, AEs, ADRs, and causality assessment within the CRF will use CRF as source data.

## PROCEDURES FOR DIRECT ACCESS

Before initiating the study, the sponsor and the principal investigator/institution will mutually decide on specific agreed-upon procedures for directly accessing the source documents. (Refer to “Procedures for Direct Access to Source Documents”)

## EVALUATION AND HANDLING OF VERIFICATION OF RESULTS

The principal investigator will provide a written explanation of any discrepant results between the CRF data and source documents to the sponsor, and retain a copy of the original.

Any confirmed deviation or noncompliance with the protocol will be promptly recorded and notified to the principal investigator by the sponsor. In addition, the sponsor will explicate appropriate protocol adherence to the principal investigator in order to secure his/her compliance.

# QUALITY CONTROL AND QUALITY ASSURANCE

## QUALITY CONTROL

In order to evaluate whether the quality of activities of the Good Clinical Practice (GCP) and statistical analysis departments and study medication administrator fulfill the necessary requirements, the sponsor`s study-related department will perform quality control in accordance with Tsumura-GCP/standard operating procedures (SOP).

## QUALITY ASSURANCE

The sponsor`s GCP audit department will conduct GCP audit according to the Tsumura-GCP SOP for Audit and based on the schedule described in the Audit SOP, to evaluate the study`s compliance with the protocol, Tsumura-GCP/SOP, Tsumura GCP and standard GCP. The GCP audit will be conducted at the institution and other study sites as well.

# PUBLICATION POLICY

The results of the study may not be presented at academic conferences or published in medical journals without the consent of the sponsor.

# STUDY PERIOD

May 2012 – December 2012

# STUDY ORGANIZATION

## SPONSOR

### Sponsor

Tsumura & Co.

2-17-11 Akasaka Minato-ku, Tokyo, 107-8521, Japan

[Contact information]

Kampo Research Planning Department

TEL: +813-6361-7185 FAX: +813-5574-6664

### Sponsor`s Study Organization

The study organization of the sponsor is described in the “Organization of Sponsor of the Post-Marketing Clinical Study of TJ-54-4-3 (Attached document 3)”.

### Medical Advisor

Department of Elderly General Medicine, Tokyo Medical University

Professor Haruo Hanyu, M.D.

[Role of the Medical Advisor (Medical Expert)]

The medical expert is an appropriately qualified medical personnel who will be readily available to advise on study related medical questions or problems. Specifically, the medical personnel will review the content of the adverse events reports received from the study department.

## TESTING FACILITY FOR TJ-54 CONSITUENTS IN PLASMA

ADME & Tox. Research Institute, Sekisui Medical Co., Ltd.

2117 Muramatsu, Tokai, Ibaraki 319-1182 Japan

TEL: +81-29-282-0232 FAX: +81-29-282-0182

## RESEARCH FACILITY AND PRINCIPAL INVESTIGATOR

Research facility: Kochi Medical School Hospital

Oko-cho, Kohasu, Nankoku-shi, Kochi 783-8505, Japan

TEL: +81-88-866-5811

Principal Investigator: Professor Kazuhiro Hanazaki, M.D., Ph.D.

First Department of Surgery, Kochi Medical School Hospital

# REFERENCES

- - - 1. Shinno H. et al. Effect of Yi-Gan San on psychiatric symptoms and sleep structure at patients with behavioral and psychological symptoms of dementia. Pro Neuro-Pharm Biol Psychi. 2009, 32, p.881-885.

1. Iwasaki K. et al. A randomized, observer-blind, controlled trial of the traditional Chinese medicine Yi-Gan San for improvement of behavioral and psychological symptoms and activities of daily living in dementia patients. J Clin Psychiatry. 2005, 66, p.248-252.
2. Iwasaki K. et al. Effects of the traditional Chinese herbal medicine Yi-Gan San for cholinesterase inhibitor-resistant visual hallucinations and neuropsychiatric symptoms in patients with dementia with Lewy bodies. J Clin Psychiatry. 2005, 66, p.1612-1613.
3. Mizukami K. et.al. A randomized crossover study of a traditional Japanese medicine (Kampo)“Yokukansan” in the treatment of the behavioral and psychological symptoms of dementia. Int. J. Neuropsychopharm. 2009, 12, p.91-99.
4. Monji A. et.al. Effect of yokukansan on the behavioral and psychological symptoms of dementia in elderly patients with Alzheimer’s disease. Pro Neuro-Pharm Biol Psychi. 2009, 33, p.308-311.
5. Ikarashi Y. et al. Effects of yokukansan, a traditional Japanese medicine, on memory disturbance and behavioral and psychological symptoms of dementia in thiamine-deficient rats. Biol Pharm Bull, 2009, 32, p.1701-1709.
6. Tabuchi M. et al. Ameliorative effects of yokukansan, a traditional Japanese medicine, on learning and non-cognitive disturbances in the Tg2576 mouse model of Alzheimer’s disease. J Ethnopharmacol. 2009, 122, p.157-162.
7. Sekiguchi K. et al. Effects of yokukansan, a traditional Japanese medicine, on aggressiveness induced by intracerebroventricular injection of amyloid β protein into mice. Phytother Res. 2009, 23, p.1175-1181.
8. Kanno H. et al. Effect of yokukansan, a traditional Japanese medicine, on social and aggressive behaviour of para-chloroamphetamine-injected rats. J Pharm Pharmacol. 2009, 61, p.1249-1256.
9. Egashira N. et al. Repeated administration of Yokukansan inhibits DOI-induced head-twitch response and decreases expression of 5-hydroxytryptamine (5-HT)2A receptors in the prefrontal cortex. Prog Neuropsychopharmacol Biol Psychiatry. 2008, 32, p.1516-1520.
10. Terawaki K. et al. Partial agonistic effect of yokukansan on human recombinant serotonin 1A receptors expressed in the membranes of Chinese hamster ovary cells. J Ethnopharmacol. 2010, 127, p.306-312.
11. Kawakami Z. et al. Neuroprotective effects of yokukansan, a traditional Japanese medicine, on glutamate-mediated excitotoxicity in cultured cells. Neurosci. 2009, 159, p.1397. -1407.
12. Kawakami Z. et al. Glycyrrhizin and its metabolite 18β-glycyrrhetinic acid in glycyrrhiza, a constituent herb of yokukansan, ameliorate thiamine deficiency-induced dysfunction of glutamate transport in cultured rat cortical astrocytes. Eur J Pharmacol. 2010, 626, p.154-158.
13. Takeda A. et al. Suppressive effect of Yokukansan on excessive release of glutamate and aspartate in the hippocampus of zinc-deficient rats. Nut Neurosci. 2008, 11, p.41-46.
14. Takeda A. et al. Attenuation of abnormal glutamate release in zinc deficiency by zinc and yokukansan. Neurochem Int. 2008, 53, p.230-235.
15. Company data of Tsumura & Co.
16. Preliminary absorption study of yokukansan (YKS:TJ-54)in healthy volunteers TJ-54 (TJ-54-4-2)
17. Kanatani H,　et al.　The active principles of the branchlet and hook of Uncaria sinensis Oliv. examined with a 5-hydroxytryptamine receptor binding assay.　 J Pharm Pharmacol. 1985, 37(6), p.401-404.
18. [Sakakibara I](http://www.ncbi.nlm.nih.gov/pubmed?term="Sakakibara I"%5BAuthor%5D), et al. Effect on locomotion of indole alkaloids from the hooks of uncaria plants. [Phytomedicine.](http://www.ncbi.nlm.nih.gov/pubmed) 1999, 6(3), p.163-168.
19. Shimada Y,et al.　Evaluation of the protective effects of alkaloids isolated from the hooks and stems of Uncaria sinensis on glutamate-induced neuronal death in cultured cerebellar granule cells from rats.　 J Pharm Pharmacol. 1999, 51(6), p.715-722.
20. Makino T. et al. Down-regulation of a hepatic transporter Mrp2 is involved in alteration of pharmacokinetics of glycyrrhizin and its metabolites in a rat model of chronic liver injury. 2008 Drug Metab. Dispos. 36(7) p1438-1443

19 Statistician and Medical Advisor (Medical Expert) Verification

【Ver. 1.0　Prepared on December 27, 2011】

Statistician 　 Month　 　Day　 　Year

Medical Advisor Month　 　Day　 　Year

20 CLINICAL TRIAL AGREEMENT

Statement of Agreement

The seals and signatures below constitute the agreement and approval of this protocol and the attachments, and provide the necessary assurances that this trial will be conducted according to all stipulations of the protocol (Ver.1.0) and Case Report Form (Ver.1.0) of the “Pharmacokinetic study of single oral administration of yokukansan (TJ-54) in healthy volunteers”.

　　Principal Investigator Month Day ２０１2

Research facility　　Kochi Medical School Hospital

Affiliation/Title　　　First Department of Surgery, Professor

　　　 Name　 Kazuhiro Hanazaki 　　　 　　　　　　　印

　　Study Monitor Month Day ２０１2

Tsumura & Co.

　　　　　 Head of Kampo Research Planning Department

　　　　　 Name　　 Kazuya Maemura　　　　　　　　　　　　　印
